# Compound drought–heat wave hazards are concentrated in US regions with multiple social vulnerabilities and agricultural dependence

**DOI:** 10.1073/pnas.2607273123

**Published:** 2026-09-21

**Authors:** Amitesh Sabut, Ashok Mishra, David Zilberman

**Affiliations:** ^a^ Zachry Department of Civil Engineering, Texas A&M University, College Station, TX 77843; ^b^ Department of Agricultural and Resource Economics, University of California, Berkeley, CA 94720

**Keywords:** compound climate extremes, socioeconomic vulnerability, climate risk inequality

## Abstract

Compound drought and heat wave (CDHW) events are becoming more frequent and intense across the contiguous United States, increasing risks to human health, ecosystems, agriculture, and energy production systems. However, their distribution across socioeconomically vulnerable communities remains largely unexplored at the national scale. In this study, we develop a CDHW metric by integrating weekly US Drought Monitor classifications with daily temperature extremes from 2000–2021, allowing consistent mapping of CDHW occurrences along county-level socioeconomic conditions. Our analysis reveals significant regional differences, with elevated CDHW occurrences concentrated in the South, Southeast, and western United States. Regions facing greater socioeconomic challenges, including poverty, high agricultural employment, and health-related vulnerabilities, experience notably higher CDHW risks. Greater spatial concentration of CDHW occurrences, as indicated by higher Gini coefficient values, occurs where multiple socioeconomic vulnerabilities intersect, such as in counties with large poor populations and older housing. These findings indicate that CDHW events are unevenly distributed across socially vulnerable communities, underscoring the need for targeted adaptation strategies to reduce climate risks for the most vulnerable communities.

## Significance

Compound drought and heat wave (CDHW) events intensify risks to health, agriculture, energy systems, and local economies, often producing more severe impacts than isolated extremes. Their growing relevance was evident during the 2023 southern US heat emergencies and widespread drought, which exposed persistent social and infrastructural weaknesses across many regions. This study maps CDHW occurrence across multiple dimensions of county-level vulnerability, including socioeconomic disadvantage, agricultural dependence, and health-related sensitivity, to identify vulnerable regions across the United States. We find that CDHW exposure is unequally distributed, with greater burdens in counties facing multiple social and infrastructural vulnerabilities; for example, counties with higher poverty levels and older housing conditions bear a disproportionate share of CDHW exposure, underscoring the need for equity-focused adaptation strategies.

Over the past four decades, climate-related disasters have caused more than $1.775 trillion in economic losses and over 14,223 deaths in the United States (1, 2). Among these hazards, droughts and heat waves stand out as significant threats (3). Although droughts and heat waves affect society through different pathways, they are strongly interconnected. Heat waves directly threaten human health through heat stress, cooling demand, and mortality risk, especially among elderly, medically dependent, outdoor-working, and low-income populations (4, 5), whereas droughts affect water availability, agricultural production, ecosystems, and food systems, with impacts extending from rural producers to urban consumers through crop losses, higher food prices, and pressure on water and energy systems (6, 7). When these hazards co-occur as compound drought and heat wave events (CDHWs), dry soils limit evaporative cooling and amplify near-surface heat, while extreme heat increases atmospheric water demand and accelerates vegetation and soil moisture stress. Therefore, CDHWs represent a coupled water–energy hazard that can simultaneously affect public health, agriculture, energy use, and local adaptive capacity, causing impacts more severe than either hazard alone, particularly during summer months when human thermoregulatory stress is greatest (8–10). Quantitative evidence from previous studies also shows that CDHW impacts can exceed those of drought or heat wave alone. In a recent US health study, heat wave-attributable mortality among patients with chronic obstructive pulmonary disease increased from 2.91% under nondrought conditions to 7.41% under drought conditions (11). Across US corn and soybean production, CDHWs reduced water-use efficiency by 14.7% and 11.3%, respectively, and caused larger yield losses than either drought or heat wave alone (12). These findings support evaluating CDHWs as joint hazards rather than independent events. In recent years, CDHW frequency and severity have escalated, posing significant risks to agriculture, ecosystems, public health, and energy infrastructures (13–16). For example, the 2022 drought and heat wave event in the western United States caused $22.1 billion in damages, while the 2023 event across the South and Midwest resulted in $14.5 billion in losses (17).

However, the impacts of compound climate hazards in the United States are not shared equally across populations. Deep-rooted racial and economic divisions continue to shape unequal exposure to environmental risks (18, 19). Socially and economically marginalized communities often face heightened vulnerability due to poor housing conditions (20), limited access to cooling (21) and healthcare (22), and financial constraints (21) that hinder their ability to adapt. Vulnerability is also rising in urban areas, driven by factors such as population density, urban heat, and land surface changes (23–25). Older adults, in particular, are physiologically more susceptible to heat stress and often lack timely assistance during extreme events, regardless of location (26, 27). As a result, analyses that focus only on physical hazard intensity, without considering social vulnerabilities, may underestimate the burden on at-risk populations (28, 29). Beyond physical exposures, unevenness itself acts as a structural driver of disaster outcomes (30), with greater income-related disparities associated with higher disaster-related mortality and reflecting persistent differences in governance, emergency services, and investments in resilience (31–33). Adaptation efforts that overlook these dimensions risk reinforcing or worsening existing disparities (34).

While the risks from CDHW events are rising, there remains a notable gap in national-scale analyses within the United States that explicitly examine how these hazards intersect with socioeconomic vulnerability. To date, no comprehensive nationwide study has systematically quantified variations in CDHW occurrence across different socioeconomic contexts or assessed how intersecting socioeconomic conditions are associated with uneven spatial patterns of risk. This gap poses significant challenges for developing equitable adaptation strategies to protect vulnerable communities.

To address this gap, we analyze CDHW events across US counties from 2000 to 2021. We quantify the average duration of summer CDHW events and examine how CDHW occurrence varies geographically and across different socioeconomic sectors. We integrate weekly drought data from the US Drought Monitor and daily temperature records from the Climate Prediction Center to identify CDHW events. Socioeconomic data reflecting economic, demographic, infrastructural, and health-related dimensions of community vulnerability are combined with these climate datasets. Using spatially varying coefficient (SVC) modeling, which allows climate–socioeconomic associations to vary across space rather than assuming one generalized CONUS-wide relationship, we assess how associations between CDHW occurrence and socioeconomic factors differ across regions (35, 36). Furthermore, we use Lorenz curves and Gini coefficients to quantify spatial heterogeneity in the distribution of CDHW occurrence across counties with varying socioeconomic characteristics (37, 38). In this context, higher Gini values indicate that CDHW occurrence is more concentrated among a smaller share of the population. Our findings provide a nationwide assessment of CDHW occurrence across vulnerable US communities and demonstrate the need for targeted adaptation strategies to reduce risk and strengthen resilience.

## Results

### Mapping County-Level Occurrences of CDHWs.

**Fig. 1.**
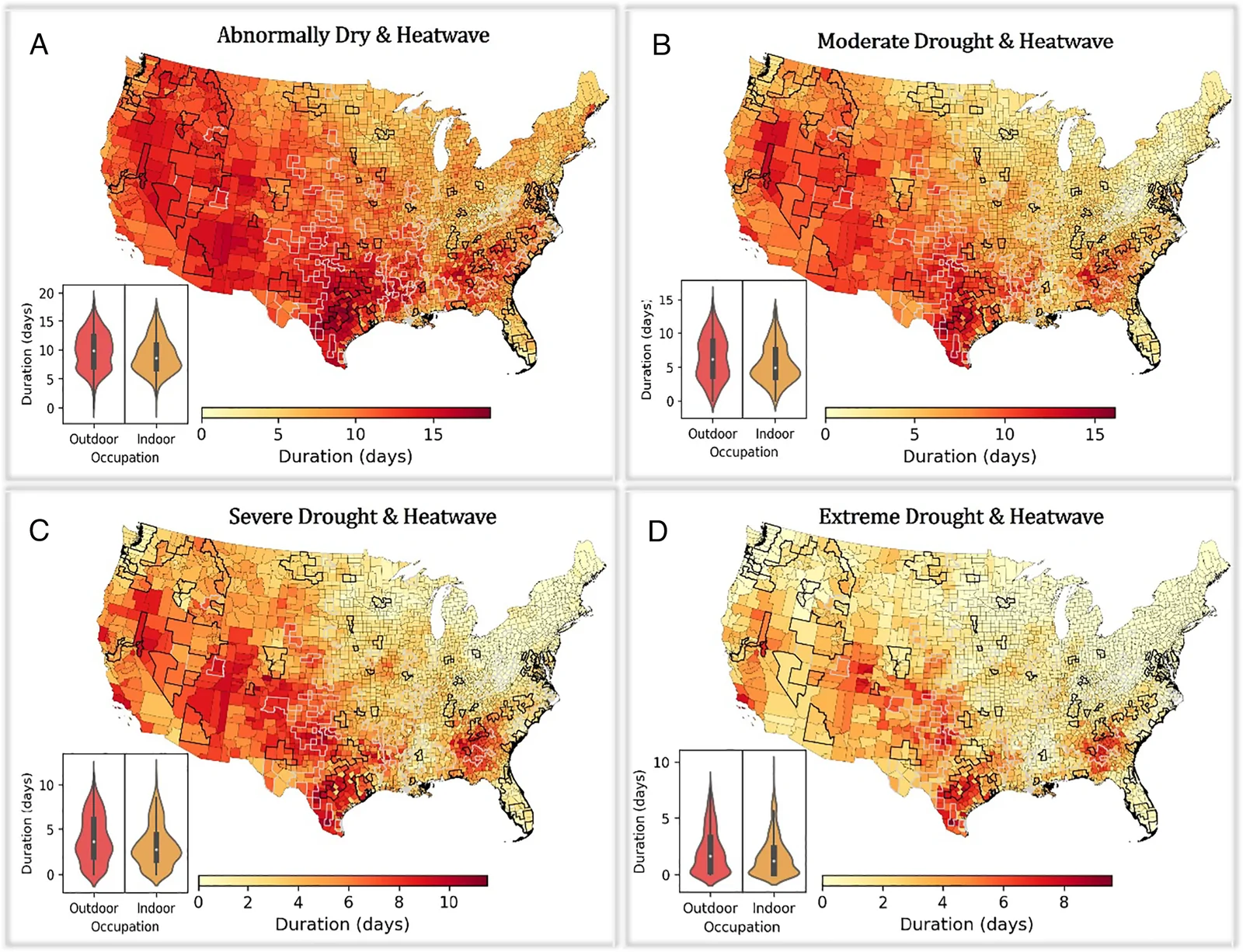
Mapping county-level occurrences of CDHWs: the plot shows the average number of days counties across the CONUS experienced CDHW events during summer (May–October) from 2000 to 2021, under four drought severity levels (*A*–*D*). Counties outlined in black indicate population increases, and those in white indicate decreases, both representing changes with magnitudes above the upper quartile. Violin plots in each panel display the distribution of CDHW durations in counties predominantly engaged in outdoor versus indoor occupations.

We analyzed the average summer-season (May–October) duration of CDHW events across counties in the Continental United States (CONUS) for 2000–2021. Here, county-level CDHW occurrence is expressed in days and represents the average number of summer days during which CDHW conditions were present, rather than the count of separate CDHW event episodes. We categorized drought severity into four levels (abnormally dry, moderate, severe, and extreme) based on US Drought Monitor classifications (39). Drought Monitor classifications. Spatial distributions of CDHW duration (Fig. 1 *A*–*D*) reveal substantial regional disparities. Under abnormally dry conditions (Fig. 1*A*), southwestern counties, particularly in Arizona and central Texas, averaged over 15 CDHW days per summer, while northern and northeastern counties generally recorded fewer than 5 d. Scattered pockets in the Southeast showed localized averages between 8 and 12 d. During moderate droughts (Fig. 1*B*), CDHW activity intensified, with the Great Basin region (between the Rockies and Sierra Nevada) and central Texas frequently exceeding 15 d, while northern regions largely remained below 5 d. Under severe drought conditions (Fig. 1*C*), CDHW durations declined nationwide, with median values falling below ~10 d, though hotspots persisted in central Texas, the Great Basin, and the Southeast, where some counties reached ~12 d. For extreme droughts (Fig. 1*D*), CDHW events became rarer and spatially limited, with only small areas in the southern Great Plains, central Texas, and parts of the Southeast recording over 8 d, while most regions averaged fewer than 3 d. Overall, the southern, southeastern, and western United States consistently experienced the highest CDHW occurrences, averaging 8 to 15 d under mild-to-moderate droughts, compared to less than 5 d in northern and northeastern regions. As drought severity intensified, CDHW events diminished in extent and frequency, though certain regions remained persistently affected, indicating localized vulnerability even under extreme conditions.

To explore links between CDHW occurrence and demographic trends, we analyzed county-level population change using US Census data from 2010 to 2020 (40). Counties in the highest and lowest quartiles of population change (>75th and <25th percentiles) are outlined in black and white, respectively, in Fig. 1 *A*–*D* (for details, see *SI Appendix*, section A.3 and Fig. S3). Counties with high population growth, defined as those in the upper quartile of population change from 2010 to 2021 (>75th percentile), were concentrated in the western United States and central Texas, while population decline was prominent across the central Great Plains and parts of the eastern United States. Under abnormally dry and moderate drought conditions, 60 to 70% of counties with high population growth overlapped with areas experiencing more than 7 CDHW days per summer, but this overlap dropped below 10% under severe and extreme droughts. In contrast, counties with declining populations showed steady exposure across all drought severities, with ~15 to 20% recording at least 7 CDHW days even during severe drought. These findings indicate that while population growth tends to coincide with moderate CDHW risk, areas with population decline remain consistently vulnerable to severe and extreme CDHW events, highlighting compounded risks in demographically challenged regions. Further, regional hypothesis testing (*SI Appendix*, section A.7 and Table S2) shows that most regions experiencing higher CDHW occurrences between 2010 and 2020 also saw significant population declines.

We further assessed occupational vulnerability using 2017–2021 American Community Survey data (41), classifying counties into two categories: outdoor occupations (farming, mining, recreation) and indoor occupations (manufacturing, government), reflecting the nature of work environments (for details, see *SI Appendix*, section A.3). Violin plots in Fig. 1 *A*–*D* illustrate CDHW durations for counties dominated by outdoor versus indoor occupations across drought severities. Across all conditions, counties with outdoor occupations consistently experienced longer CDHW durations. Under abnormally dry and moderate droughts, median durations in outdoor-occupation counties ranged from ~6 to 8 d, sometimes exceeding 15 to 20 d, while indoor-occupation counties showed lower medians (~4 to 5 d). As drought severity increased, median durations declined to ~3 to 5 d for outdoor occupations and ~2 to 3 d for indoor occupations. However, outdoor occupations maintained broader distributions and longer upper tails, indicating higher variability and a greater likelihood of prolonged CDHW occurrences.

### Patterns of CDHW Occurrences Across Socioeconomic Vulnerabilities.

**Fig. 2.**
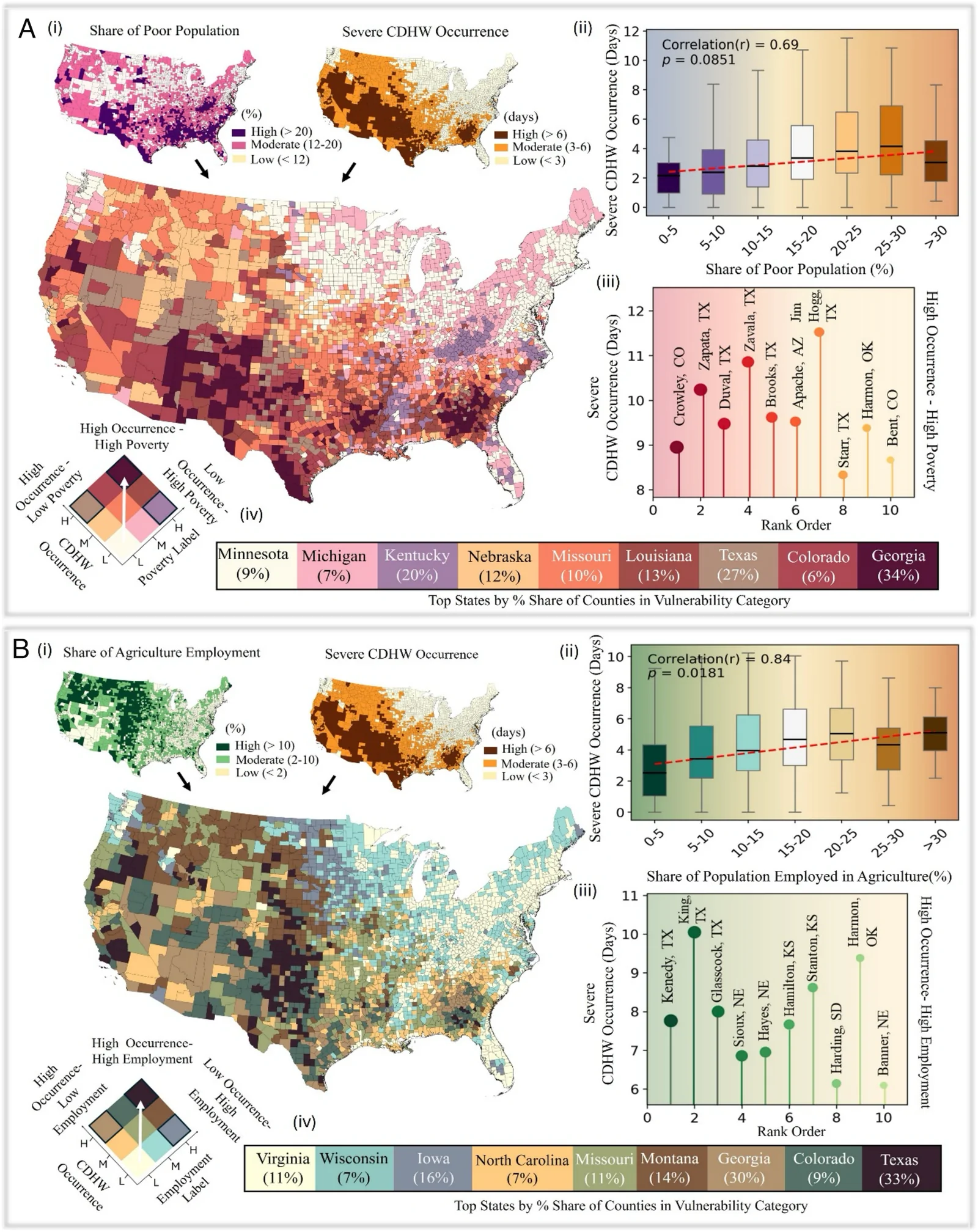
Patterns of severe CDHW occurrences across socioeconomic vulnerabilities: Panel (*A*) shows severe CDHW exposure in high-poverty and (*B*) in high-agricultural-employment counties, each showing: (*i*) a bivariate choropleth map showing the spatial overlap; (*ii*) boxplots show the relationship between poverty rate and CDHW occurrence, while the red line indicates the fitted linear regression between poverty rate (x-axis) and median CDHW occurrence; (*iii*) the 10 most vulnerable counties; and (*iv*) top states’ contributions to each bivariate vulnerability category.

We analyzed severe CDHW Occurrences in relation to county-level socioeconomic conditions across the continental United States, focusing on poverty rates and agricultural employment. Although CDHW events were assessed across four severity levels, we focused on severe events due to their significant impacts and broader spatial coverage suitable for statistical analysis. Fig. 2 *A, i* maps counties by poverty levels categorized as low (<12%), moderate (12 to 20%), and high (>20%), and overlays them with severe CDHW days classified as <3, 3 to 6, and >6 d. High-poverty counties cluster across the South, Appalachia, and parts of the Southwest, while severe CDHW days are most prevalent in the Southwest and Southern Plains. Overlaying these maps reveals vulnerable corridors, particularly from the Southern Plains through the Deep South, where high poverty aligns with frequent severe CDHW events. Regional contrasts are also evident, with some western and Great Basin region experiencing highly severe CDHW days despite low poverty, while areas like Appalachia and the Gulf Coast remain socioeconomically disadvantaged yet see fewer severe CDHW days.

Fig. 2 *A, ii* shows median severe CDHW days increasing across poverty quantiles, with a positive association (r = 0.69, *P* = 0.0851), suggesting higher poverty often coincides with elevated climate stress. Among counties with both high poverty and high severe CDHW occurrence, Jim Hogg County, Texas, recorded the highest average severe CDHW duration (~11.5 d), followed by other Texas counties (~ 10 to 10.5 d), and Apache County, Arizona (~9.5 d). At the state level, Georgia had the largest share of counties facing both high poverty and high severe CDHW (34%). Conversely, Texas had many counties with high severe CDHW but low poverty (27%), while Kentucky led in low severe CDHW but high poverty (20%) (Fig. 2 *A, iv*).

Agricultural employment showed a similarly strong association with severe CDHW occurrences. Fig. 2 *B, i* maps counties by agricultural workforce share (low <2%, moderate 2 to 10%, and high >10%) alongside severe CDHW levels. High agricultural employment was most prominent in the Great Plains, Midwest, and parts of the West, often overlapping with severe CDHW hotspots. Counties where over 10% of the workforce is in agriculture face dual risks from economic dependence on farming and climate extremes, though this relationship varies regionally. For instance, the Southwest experiences high severe CDHW despite low agricultural employment, while some northern Great Plains and Midwest regions have high agricultural employment yet relatively few severe CDHW days. Fig. 2 *B, ii* shows a strong positive association (r = 0.84, *P* = 0.0181), with median severe CDHW days increasing alongside agricultural employment. Among counties with both high agricultural employment and severe CDHW, King County, Texas, recorded ~10.2 d, while Glasscock and Kenedy counties reported 7.8 to 9.5 d. Stanton County, Kansas, and Sioux County, Nebraska, experienced ~8 to 8.5 d. At the state level, Texas led with 33% of its counties in the high-agriculture, high-severe CDHW category. Conversely, Georgia had the highest share (30%) of counties with high severe CDHW but low agricultural employment, while Iowa topped the list for low severe CDHW but high agricultural employment (16%) (Fig. 2 *B, iv*).

Beyond poverty and agriculture, other socioeconomic variables were significantly associated with severe CDHW occurrences (*SI Appendix*, Fig. S5). Higher rates of severe CDHW were observed in counties with greater shares of residents lacking a high school diploma (r = 0.91, *P* = 0.00419), medically dependent populations (r = 0.94, *P* = 0.00544), uninsured individuals (r = 0.80, *P* = 0.0304), and populations reporting delayed emergency medical care (r = 0.49, *P* = 0.259). Conversely, severe CDHW occurrences were negatively correlated with older housing (r = −0.75, *P* = 0.0513) and low food access (r = −0.87, *P* = 0.0106), reflecting regional development and urban–rural differences. Severe CDHW occurrences showed only a weak association with elderly population share (r = 0.21, *P* = 0.644). Collectively, the findings show that socioeconomic conditions are closely associated with the geographic patterns and intensity of severe CDHW events across the CONUS, highlighting significant differences in occurrences among regions and communities.

### Regional Heterogeneity in CDHW Occurrence and Socioeconomic Vulnerabilities.

**Fig. 3.**
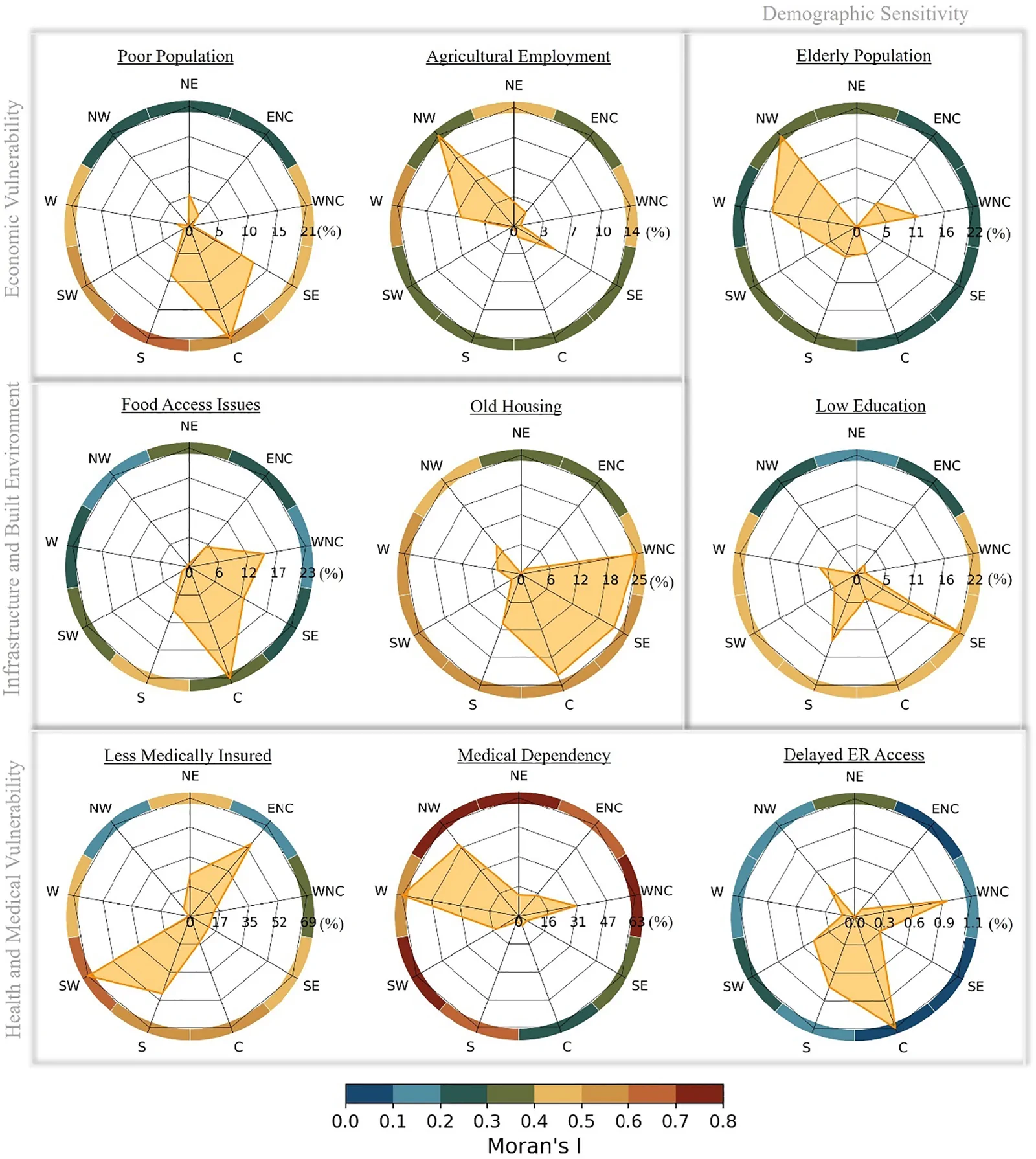
Regional heterogeneity in CDHW occurrence and socioeconomic vulnerabilities. Radar plots show the average percentage of severe CDHW exposure spatially associated with each socioeconomic factor across nine US climate regions, based on SVC modeling. The outer ring shows Moran’s I, with higher values indicating stronger clustering and lower values indicating greater spatial dispersion across regions.

To examine regional heterogeneity in how socioeconomic vulnerabilities are spatially associated with severe CDHW Occurrence across US counties, we used a SVC modeling framework estimated via integrated nested Laplace approximation (INLA). For each county, we calculated the percentage association of each socioeconomic variable with severe CDHW occurrence, considering only positive associations. These values were then averaged across nine predefined US regions to highlight regional differences in the strength of these associations, as shown in the radar plots in Fig. 3. Along the outer ring of each radar plot, we display regional Moran’s I values, which quantify the degree of spatial clustering of each socioeconomic variable within that region. Higher Moran’s I values indicate stronger spatial clustering (i.e., counties with similar vulnerability levels are geographically grouped), while lower values indicate more spatial dispersion (*SI Appendix*, section A.5 for details, *SI Appendix*, Fig. S8).

The results reveal pronounced regional differences in how socioeconomic conditions align with severe CDHW occurrences. In the Central region, poverty (20.6%), limited food access (23.1%), and delayed emergency room availability (7%) each account for a notable share of the local spatial association with severe CDHW occurrences. Their Moran’s I values of approximately 0.50, 0.30, and 0.10 indicate that poverty occurs in well-defined clusters, food access limitations form moderately grouped areas, and emergency service delays are broadly scattered across counties. In the Southeast, old housing and low educational attainment represent 21 to 25% of the local association, followed by poverty at 12%. All three variables exhibit Moran’s I values near 0.50, indicating clear regional clusters of structural and socioeconomic disadvantage. In the Southwest, lack of medical insurance represents 69.0% of the association with severe CDHW occurrence, and Moran’s I values between 0.60 and 0.70 show that uninsured populations are concentrated in tightly grouped areas.

The western regions display a different configuration. In the Northwest, elderly populations (21.9%) and agricultural employment (13.7%) explain comparable portions of the local association with severe CDHW occurrence. Their Moran’s I values (0.20 to 0.30) indicate that these vulnerabilities are distributed across many counties, with only limited pockets of concentration. In the West, medical dependency (63.0%) and elderly populations (16.0%) account for much of the regional association, and their Moran’s I values (0.20 to 0.40) suggest that these conditions are distributed widely rather than concentrated in sharply defined clusters. In the Western North Central region, old housing accounts for about 20% of the spatial association and exhibits moderate clustering (Moran’s I ~0.40), whereas limited food access accounts for about 13% and displays a highly dispersed pattern (Moran’s I ~0.10). These contrasts show that regions vary not only in which socioeconomic factors align with severe CDHW occurrence but also in how geographically organized those vulnerabilities are, shaping the magnitude and spatial intensity of regional risk.

### Concentration of CDHW Occurrence across Intersecting Socioeconomic Vulnerabilities.

**Fig. 4.**
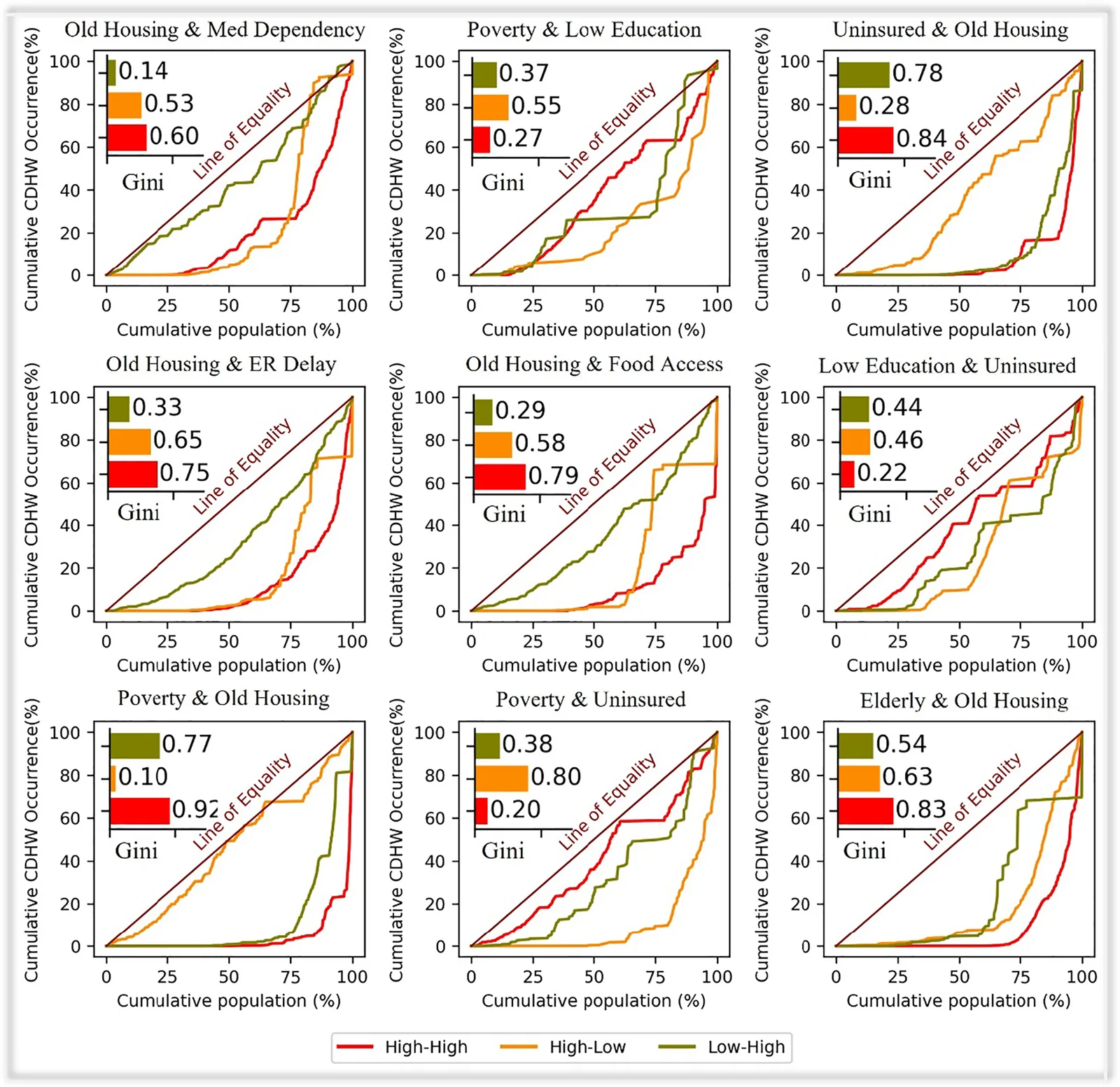
Concentration of CDHW occurrence across intersecting socioeconomic vulnerabilities. Lorenz curves show the cumulative distribution of severe CDHW occurrences across counties in the CONUS for specific pairs of socioeconomic vulnerabilities. For each pair, counties are grouped into High–High, High–Low, and Low–High categories based on the levels of both vulnerabilities. The diagonal maroon line indicates perfect equality, where CDHW occurrences are evenly distributed across the population. Gini coefficients indicate the degree of unevenness, with higher values reflecting more concentrated exposure among a smaller portion of the population.

To examine how severe CDHW occurrence is concentrated across intersecting socioeconomic conditions, we analyzed paired combinations of socioeconomic vulnerability indicators across US counties. Each vulnerability indicator was classified into three levels: high, moderate, and low, based on its relative ranking among all counties. We then combined two indicators at a time to form paired vulnerability groups, such as High–High (e.g., counties with both high poverty rates and a large elderly population), High–Low, and High–Moderate, and other combinations. This categorization allowed us to assess how the distribution of CDHW occurrences differs across varying levels and combinations of socioeconomic vulnerability (*SI Appendix*, section A.6). Using Lorenz curves and Gini coefficients, we identified which pairings exhibited the greatest disparities in severe CDHW occurrences. Fig. 4 highlights the top nine vulnerability pairs with the largest differences in occurrence between High–High and Low–Low counties (*SI Appendix*, Fig. S9), while Gini coefficients for all combinations across all vulnerability groups are shown in *SI Appendix*, Fig. S10.

Our analysis shows that CDHW occurrence is not evenly distributed across counties; rather, it is spatially concentrated among counties where multiple socioeconomic vulnerabilities overlap. A higher Gini coefficient indicates greater unevenness in CDHW occurrence, where a smaller number of counties account for a larger share of CDHW events within that vulnerability grouping. For instance, the old housing–medical dependency pairing, CDHW occurrences are highly concentrated in counties where both vulnerabilities are elevated (Gini = 0.60) and remain substantial when housing vulnerability is high alongside moderate medical dependency (0.53), whereas low housing vulnerability aligns with far lower concentration (0.14). Moderate poverty combined with high educational vulnerability also shows notable concentration (Gini = 0.55). The uninsured–old housing combination presents some of the sharpest disparities, with Gini values of 0.84 where both vulnerabilities are high and 0.78 where housing vulnerability alone remains elevated. Old housing paired with delayed emergency room access records a concentration level of 0.75 in high-vulnerability counties. Old housing combined with food access barriers reaches 0.79 where both are high but declines to 0.29 when only housing vulnerability is elevated. For elderly populations and old housing, concentration remains consistently elevated across configurations, with Gini values of 0.83 where both vulnerabilities are high, 0.63 where elderly vulnerability is high, but housing vulnerability is low, and 0.54 where housing vulnerability is high but elderly vulnerability is low.

Across these groupings, CDHW occurrences are distributed unevenly, with a small segment of the population experiencing a disproportionate share of events when multiple vulnerabilities coincide. In counties characterized by elevated poverty levels combined with aging or substandard housing, the most affected 20% of residents account for nearly 80% of total CDHW occurrences (Gini = 0.92), demonstrating a steep concentration in communities facing sustained socioeconomic challenges. Comparable levels of concentration are observed where housing-related vulnerabilities intersect with food access limitations, delayed emergency care, or a high proportion of uninsured residents, indicating that deteriorating housing conditions persist as a central and recurring element across multiple unequal configurations.

Triplet combinations with high levels across all three vulnerabilities revealed even more pronounced disparities in CDHW occurrences (*SI Appendix*, Fig. S11). Counties with high poverty, low education, and old housing record the highest Gini value (0.95), followed by elderly population, old housing, and food access barriers (0.93), and poor population, elderly population, and old housing (0.91). The combination of poverty, old housing, and food access issues also shows elevated concentration (Gini = 0.85). These patterns indicate that when socioeconomic vulnerabilities stack, particularly those related to constrained incomes, aging populations, and substandard housing, the distribution of CDHW occurrences becomes highly uneven, with a disproportionate share concentrated within a limited number of structurally vulnerable counties.

## Discussion and Implications

In this study, we developed a high-resolution, county-scale CDHW metric by integrating weekly drought indices with daily temperature data to map CDHWs across the CONUS from 2000 to 2021. Distinct regional hotspots emerged in the West, South, and Southeast, each shaped by different physical and socioeconomic vulnerabilities. In the West, dependence on snowpack, limited water availability, and reliance on hydropower heighten risks to water and energy systems (42, 43). In the South, large urban populations and intensive agriculture amplify exposure through elevated water stress, energy demand, and crop losses (44). The Southeast faces added health and infrastructure strain from extreme humidity during heat–drought periods (45, 46). These patterns align with previous evidence of worsening drought in the West and increasing heat extremes in the Southeast (47, 48). Future projections indicate that longer and more frequent CDHWs will intensify these regional disparities (49), particularly for low-income and marginalized populations (3, 50).

Regional hypothesis testing shows that most areas with rising CDHW occurrences are experiencing population decline. However, the Northeast and the southern Gulf Coast are exceptions: despite increasing CDHW occurrences, both regions continue to grow, which may be linked to economically resilient metropolitan centers and sustained retirement migration (51, 52). This pattern reflects a nonlinear amenity–risk transition; moderate warming may still attract residents to high-opportunity regions, whereas persistent heat stress contributes to population decline (53), weakening the long-standing Sunbelt migration advantage (54). Structural disparities further shape these outcomes, as poor insulation, mobile homes, and heat-intensive outdoor work heighten vulnerability (55, 56), whereas newer housing and adaptive farm practices provide only partial protection (57). Institutional conditions play a defining role, with counties facing high uninsured rates, healthcare shortages, or weak heat-standard enforcement that face greater illness and mortality risks, especially among agricultural workers (58).

The spatial pattern of CDHW occurrence mirrors broader socioeconomic and demographic divides. Counties with higher poverty rates show markedly greater occurrences, particularly in the Southeast, Southwest, and Deep South, where structural disadvantage coincides with elevated risk. In the Southeast, poverty limits adaptive capacity (18); in the Southwest, marginalization increases exposure among Tribal and Native communities (59); and along the US–Mexico border, social exclusion intensifies vulnerability for migrant and Hispanic populations (60). Similarly, high-CDHW occurrence aligns with regions of intensive agricultural employment, most evident in the Central Great Plains. Notably, several counties in these areas have experienced population declines between census periods, suggesting that CDHW events, combined with limited economic diversification, may influence agricultural livelihoods and contribute to out-migration, a pattern observed globally where climate extremes reshape rural vulnerability (61, 62). This is further reinforced by recent evidence showing that agricultural workers face rising exposure to extreme humid heat during peak planting and harvesting seasons, increasing risks to health and productivity (63), consistent with heat-driven productivity losses reported across agrarian regions worldwide (64).

Socioeconomic and demographic indicators reinforce these disparities. Counties with lower educational attainment, larger elderly populations, higher medical dependency, and limited insurance are more likely to face severe CDHW impacts because these factors restrict access to cooling, healthcare, and adaptive technologies (65). Populations with limited education are often employed in outdoor, physically demanding jobs, increasing heat exposure (66); While elderly and medically dependent individuals face heightened physiological vulnerability during heat waves, especially when compounded by power outages (67, 68). Emerging mortality projections show that adaptation to extreme heat will remain uneven, with medically vulnerable and low-income groups experiencing the greatest harm (69). Importantly, similar levels of climatic exposure do not translate into similar risk, because communities differ in their capacity to buffer impacts through access to cooling, healthcare, insurance, and protective infrastructure (70, 71). Consequently, effective CDHW risk remains higher in resource-constrained communities even under comparable climatic exposure.

Some socioeconomic indicators showed weak or nonsignificant CONUS-wide correlations with severe CDHW occurrence, particularly delayed emergency access and elderly population share, because their spatial distributions do not always align with the strongest CDHW hotspots. For example, high-CDHW urban areas around Phoenix may have stronger emergency-service infrastructure. In contrast, remote rural counties, such as parts of Montana, may have delayed emergency access but lower CDHW exposure. Similarly, elderly populations are often concentrated in retirement destinations or coastal regions, such as Florida and the East Coast (72), which may not fully overlap with the Southern CDHW hotspots. Thus, weak national correlations do not mean these factors are unimportant; their effects may be more local, context-dependent, and related to impact severity. Two distinct vulnerability patterns emerge. The Central and Southwest regions show tightly clustered socioeconomic disadvantages, while the Northwest and East North Central regions display more localized and weakly concentrated patterns. These differences vary by indicator; for example, uninsured rates align with CDHW occurrence in both regions but form tight clusters in the Southwest and are more dispersed in the East North Central region. The former require long-term structural investments, whereas the latter need targeted county-level strategies. These findings confirm that climate risk is socially shaped and spatially uneven, underscoring the need for adaptation efforts that reflect regional economic and institutional contexts (73, 74). National-scale analyses similarly find that climate damages concentrate in regions with lower adaptive capacity and weaker infrastructure (75). The paired and triplet vulnerability results further show that CDHW occurrence is most spatially concentrated where multiple vulnerabilities co-occur, including poverty, elderly populations, older housing, food access barriers, medical dependency, and delayed emergency access. These overlapping regimes suggest that CDHW risk is not driven by a single vulnerability factor, but by the spatial convergence of housing, health, infrastructure, and socioeconomic constraints. For example, older housing can increase indoor heat exposure, while elderly or medically dependent residents may be more sensitive to heat and more dependent on electricity, cooling, and timely medical care. When such conditions coincide with high poverty or limited healthcare access, CDHW exposure becomes a multisectoral risk rather than a purely climatic hazard. Recent studies further show that hydroclimatic extremes can be connected across time and space, reinforcing the need to view CDHW exposure as part of a broader cumulative-risk context rather than as an isolated local hazard (76, 77).

Translating these vulnerability-regime insights into policy demands coordinated strategies that reduce physical exposure while addressing the social conditions that intensify vulnerability. Adaptation should prioritize cooling the built environment and expanding social protection within an equity-centered framework (78). In counties where older housing, poverty, elderly populations, or medical dependency overlap, housing retrofits such as cool roofs, insulation upgrades, and reflective materials should be paired with affordable cooling support, medical-rate utility discounts, and protections against summer utility shutoffs. Urban greening and reflective materials should be prioritized in heat-burdened urban neighborhoods, particularly historically marginalized areas with low tree canopy, extensive paved surfaces, older housing, and limited access to cooling resources (79, 80). In agricultural regions with high CDHW occurrence and socioeconomic vulnerability, heat-safety rules, adjusted farm schedules, reliable shade and hydration, and mechanization of high-heat tasks should be coordinated with water planning, income support, and healthcare access for seasonal and outdoor workers (81). Expanding affordable cooling through energy assistance and protections against summer utility shutoffs can safeguard medically vulnerable groups (82), while healthcare facilities should incorporate heat-risk screening, continuity planning for electricity-dependent medical equipment, and emergency planning (83). Strengthening rural resilience requires heat-safe farm practices, improved water and income support for seasonal workers, and mobility or housing protections for medically fragile residents (12). For agricultural and rural counties, these efforts should also include drought-specific measures such as irrigation scheduling, soil-moisture monitoring, cover cropping, mulching, reduced tillage, drought-tolerant crop varieties, and local water-allocation planning (84, 85).

While our analysis offers insights into the spatial and social dimensions of CDHW exposure, several limitations should be noted. This study focuses on the spatial distribution of CDHW occurrence rather than directly observed impacts such as mortality, morbidity, crop losses, or economic damages; therefore, our findings should be interpreted as identifying spatial unevenness in climate exposure rather than realized harm. Assigning nationally consistent county-level monetary damages to CDHW events remains challenging because impacts occur through multiple pathways, including heat-related illness and mortality, agricultural losses, water shortages, increased energy demand, labor productivity reductions, and infrastructure stress. These outcomes are not consistently reported across counties and often differ in spatial scale, temporal resolution, and sectoral coverage. Therefore, Lorenz curves and Gini coefficients in this study are used to evaluate spatial heterogeneity in the distribution of CDHW occurrences across vulnerable communities, rather than to estimate realized monetary damages. Although prior research has demonstrated strong links between CDHW exposure and adverse health (10), agricultural (12), and economic outcomes (16), the translation of exposure into impacts depends on adaptive capacity, institutional response, and behavioral adjustment (86), which are beyond the scope of the present analysis. Socioeconomic indicators are treated as largely time-invariant structural characteristics, reflecting persistent conditions rather than short-term adaptation or migration responses; similarly, Old Housing is used only as a proxy for housing-related heat vulnerability because housing age may not fully capture housing quality or cooling access. County-level aggregation may also obscure finer-scale heterogeneity within urban neighborhoods or large rural counties, potentially underestimating localized vulnerability. In addition, Voronoi gap filling may smooth local CDHW variability and influence SVC associations; therefore, CDHW–socioeconomic spatial patterns should be interpreted as broad regional patterns rather than precise county-level boundaries.

Moreover, while SVC models capture regional heterogeneity in associations between CDHW occurrence and socioeconomic conditions, these relationships remain associational rather than causal and may partly reflect historical settlement patterns, land use, and climate geography. Because the SVC analysis retained only positive local associations, the coefficients should be interpreted as indicators of where socioeconomic vulnerability spatially aligns with higher CDHW occurrence, rather than as structural drivers of risk. Another limitation is that the regional contribution summaries rely on posterior mean beta values from the Bayesian SVC model and describe broad exposure–vulnerability association patterns; future studies should propagate posterior uncertainty more fully when using these estimates for county-level prioritization or targeted adaptation planning. This approach does not evaluate negative or potentially mitigating associations, and future studies should examine both positive and negative spatial relationships, ideally with outcome data and longitudinal or quasi-experimental designs, to better distinguish where socioeconomic conditions increase risk, reduce risk, or vary across regions. Despite these limitations, our findings show that CDHWs reflect underlying social and economic disparities across the CONUS and disproportionately burden resource-constrained communities. As compound extremes become more frequent (87), regionally tailored and equity-sensitive adaptation strategies will be essential to ensure that vulnerable populations are not left behind (88, 89).

## Materials and Methods

### Data Sources.

Weekly drought severity data for 2000–2021 were obtained from the US Drought Monitor gridded dataset, available through Dryad (1), and daily maximum and minimum 2-m air temperatures for the same period were sourced from the Climate Prediction Center at 0.25° resolution (2). Socioeconomic variables, including poverty levels, employment in agriculture, elderly population proportions, total population counts, population change from 2010 to 2021, occupational composition, and educational attainment, were derived from the 2017–2021 American Community Survey and compiled through the USDA Atlas of Rural and Small-Town America (https://www.ers.usda.gov/data-products/atlas-of-rural-and-small-town-america). County-level estimates of individuals reliant on electricity-dependent medical devices, such as oxygen concentrators and ventilators, were obtained from the US Department of Health and Human Services emPOWER Program as of April 2024 (https://empowermap.hhs.gov). Housing age, based on the share of structures built before 1950, and insurance coverage, including rates of uninsurance, were extracted from American Community Survey (ACS) data accessed through the US Census Bureau data portal (https://data.census.gov). Hospital facility information, including geographic location and emergency care availability, was retrieved from the April 2025 release of the CMS Hospital General Information dataset (https://data.cms.gov/provider-data/search?pagesize=50&theme=Hospitals). Regional comparisons were conducted using the National Centers for Environmental Information classification of the CONUS into nine climate regions (3). Details on data sources are provided in the Data, Materials, and Software Availability section, and the rationale for variable selection is presented in *SI Appendix*, section A.1.

### Identification of CDHWs.

CDHW events were identified by integrating weekly drought classifications from the US Drought Monitor (USDM) with daily maximum temperature data from the Climate Prediction Center (CPC). As an operational product that integrates diverse hydrometeorological indicators with field observations and expert analysis, the USDM provides high-resolution weekly assessments of drought severity. Compared to traditional indices, it more effectively captures both agricultural and hydrological drought conditions (39, 90), making it particularly well-suited for detecting short-lived compound extremes (91). Drought severity was classified using the USDM’s five-category scale (D0–D4), and a grid cell was considered drought-affected if it fell into category D0 (abnormally dry) or higher. Counties were marked drought-affected if at least 75% of their grid cells met this criterion. To address missing county-level data, spatial interpolation was applied using Voronoi tessellation based on the centroids of counties with valid data, implemented using the scipy.spatial.Voronoi function in Python (*SI Appendix*, section A.2). Heat wave days were identified when daily maximum temperature exceeded the local 90th percentile of the 2000–2021 summer climatology, derived from CPC’s Global Unified Temperature dataset. To match temporal scales, weekly drought data were disaggregated to daily resolution by assigning the same classification to all days within a given week. CDHW days were identified where drought and heat wave conditions co-occurred on the same day, mathematically expressed as

[1] $${\mathrm{CDHW}}_{i}=\left\{\begin{array}{cc} 1, & \mathrm{if}(D_{i}=1\cap H_{i}=1)\\ 0, & \mathrm{Otherwise} \end{array}\right.,$$

where $H_{i}$ and $D_{i}$ are binary indicators representing the presence of heat wave and drought conditions, respectively, on day i. A CDHW event was defined as a minimum of three consecutive CDHW days, with events separated by at least four CDHW-free days, following prior co-occurrence frameworks (8). Furthermore, CDHW days were categorized into four severity levels based on the overlapping drought classification: i) D0 + heat wave, ii) D1 + heat wave, iii) D2 + heat wave, and iv) D3/D4 + heat wave. Duration, defined as the number of consecutive CDHW days, was computed for each event and used to estimate the average summer duration for each year across the four severity levels. A schematic of the full methodological workflow, including data sources, processing steps, and definitions, is provided in *SI Appendix*, Fig. S1.

### Modeling Spatial Associations between CDHW Occurrences and Socioeconomic Vulnerability.

To analyze spatial associations in the relationship between severe CDHW occurrences and socioeconomic factors across the CONUS, we employed a Bayesian SVC modeling framework. An SVC model extends a standard regression framework by allowing regression coefficients to vary smoothly over space, rather than remaining constant across locations. In this study, the SVC model captures how the spatial association between severe CDHW occurrences and socioeconomic predictors differs across US counties, enabling the detection of regional patterns in vulnerability. We estimated this model using INLA, a Bayesian inference method that provides fast and accurate posterior estimates for latent Gaussian models without relying on computationally intensive sampling techniques such as Markov Chain Monte Carlo (92, 93). The analysis was conducted in R using the R-INLA package (https://www.r-inla.org/). Technical details on model specification, priors, and spatial structures are provided in *SI Appendix*, section A.4.

We modeled the total number of severe CDHW days in each county i as a function of nine standardized socioeconomic predictors $x_{ik}$, grouped into four domains: economic vulnerability, demographic sensitivity, infrastructure and built environment vulnerability, and health and medical vulnerability (see *SI Appendix*, Table S1). The response variable $Y_{i}$ was modeled as

[2] $$Y_{i}=\beta_{0}+\sum_{k=1}^{9}x_{\mathrm{ik}}\text{.}f_{k}\left(s_{i}\right)+f_{\mathrm{iid}}\left(i\right)+\epsilon_{i},$$

where $f_{k}\left(s_{i}\right)$ denotes the SVC for variable k at location $s_{i}$, $f_{iid}\left(i\right)$ captures county-level random effects not explained by spatial dependence, and $\epsilon_{i}$ is the residual error term.

To quantify spatial associations between socioeconomic factors and severe CDHW occurrences at each location, we computed a spatially explicit signal for each variable:

[3] $$\mathrm{Local}\;{\mathrm{association}}_{\mathrm{ik}}=\left\{\begin{array}{cc} \beta_{k}\left(S_{i}\right)\times x_{\mathrm{ik}} & \mathrm{if}\beta_{k}\left(S_{i}\right)>0\\ 0 & \mathrm{Otherwise} \end{array}\right..$$

Only positive values were retained, focusing the analysis on locations where higher vulnerability levels align with higher severe CDHW occurrences. For each county, we summed up all positive signals to obtain a total spatial association value. The local association signal for each predictor was expressed as a percentage of this total, providing a measure of the relative share of each socioeconomic factor in the county’s overall positive spatial association with severe CDHW occurrences. These percentages reflect the proportion of the county’s CDHW occurrences spatial association attributable to each socioeconomic factor, rather than causal contributions. A schematic of the full modeling workflow is provided in *SI Appendix*, Fig. S2.

### Assessment of Spatial Heterogeneity in CDHW Occurrences Across Vulnerability Groups.

We assessed spatial heterogeneity in severe CDHW occurrence across different socioeconomic contexts by constructing Lorenz curves and calculating Gini coefficients, following approaches previously used to examine uneven spatial patterns in flood risk and rainfall distribution (94, 95). We evaluated spatial heterogeneity for each socioeconomic variable and also examined all 36 unique pairwise socioeconomic variable combinations. For each pair, both variables were divided into terciles (low, moderate, and high) based on the 33rd and 66th percentiles, resulting in nine joint vulnerability categories (e.g., Low–Low, Low–Moderate, …, High–High). To evaluate unevenness within each group, counties within each socioeconomic category were sorted in ascending order based on the number of severe CDHW occurrences. For each ranked county r, we calculated the cumulative share of population and the cumulative share of severe CDHW occurrences using

[4] $$P_{r}=\frac{\sum_{j=1}^{r}p_{\mathrm{opj}}}{\sum_{j=1}^{n}{\mathrm{pop}}_{j}};\;Q_{r}=\frac{\sum_{j=1}^{r}\mathrm{CDHW}(j)}{\sum_{j=1}^{n}\mathrm{CDHW}(j)},$$

where ${\text{Pop}}_{j}$ is the population of the county j, ${\text{CDHW}}_{j}$ is the number of severe CDHW occurrences, and n is the number of counties in the category. The Lorenz curve was constructed by plotting $P_{r}$ (x-axis) against $Q_{r}$ (y-axis), with the 45° line representing perfect equality. Greater curvature below this line indicates a more unequal distribution of severe CDHW occurrences across the population. The Gini coefficient was calculated from the Lorenz curve through trapezoidal integration:

[5] $$G=1-\sum_{r=1}^{n-1}\left(P_{r+1}-P_{r}\right)\left(Q_{r+1}+Q_{r}\right),$$

where P_r_ and Q_r_ represent the cumulative shares of the population and CDHW occurrences, respectively. where $P_{r}$ and $Q_{r}$ represent the cumulative shares of population and severe CDHW occurrences, respectively. We extended this framework to triplet combinations of indicators by assigning counties to joint terciles across three variables, allowing us to assess how multidimensional socioeconomic conditions are associated with spatial heterogeneity in CDHW occurrence (*SI Appendix*, section A.6).

## Supplementary Material

Appendix 01 (PDF)

## Data Availability

All data used in this study are publicly available from the sources described in the Materials and Methods section. Processed data generated in this study are available on Zenodo. County-level CDHW occurrence data for 2000–2021 are available at https://doi.org/10.5281/zenodo.22166403 (96), and processed CDHW characteristics and emergency-room proximity data are available at https://doi.org/10.5281/zenodo.22166874 (97). Detailed county-level social vulnerability and bivariate CDHW risk data are available for download and visualization at https://amiteshsabut.github.io/US-CDHW-Social-Vulnerability/ (98).
