# Supplementary material for: Compound drought–heat wave hazards are concentrated in US regions with multiple social vulnerabilities and agricultural dependence
Source: Proc Natl Acad Sci U S A. 2026 Sep 21;123(40):e2607273123. doi: 10.1073/pnas.2607273123 (PMC13643300; doi:10.1073/pnas.2607273123)
Supplement: Supplementary file 1 [file pnas.2607273123.sapp.pdf]

Supporting Information for

Compound Drought–Heatwave Hazards Are Concentrated in U.S. Regions with Multiple Social Vulnerabilities and Agricultural Dependence

Amitesh Sabut[1], Ashok Mishra[1] and David Zilberman[2]

[1]Zachry Department of Civil Engineering, Texas A&M University, College Station, TX, USA. [2]Department of Agricultural and Resource Economics, University of California, Berkeley, CA, USA.

Corresponding Author: David Zilberman
Email: zilber11@berkeley.edu

This PDF file includes:

Appendix A.1 to A.9

Supplementary Figures S-1 to S-15

Supplementary Table 1 & 3

Data, Materials, and Software Availability Statement

SI References

Appendix A

A.1 Description and Estimation of Socioeconomic Variables

This study integrated multiple publicly available datasets to characterize county-level socioeconomic vulnerability across the continental United States. The following variables were included, with details on whether values were directly extracted or derived through computation:

- Poor Population (% below poverty line): Directly obtained from the American Community Survey (ACS) 2017–2021 5-Year Estimates via the USDA Atlas of Rural and Small-Town America.
- Agricultural Employment (% employed in agriculture): Extracted directly from the ACS 5-Year Estimates (2017–2021), summarizing the proportion of the civilian workforce employed in the agriculture sector. Available through the USDA Atlas.
- Elderly Population (% aged 65 and older): Directly taken from ACS Table B01001, representing the share of the county's total population aged 65 or above.
- Low Education (% adults without high school diploma): Obtained from ACS Table B15003, which reports educational attainment among adults aged 25 and older.
- Old Housing (% of housing units built before 1950): Extracted from ACS Table B25034 ("Year Structure Built") by summing housing units built before 1950 and dividing by total housing units in the county. This variable is used as a proxy for older building stock and potential structural housing vulnerability. Older or under-

insulated homes may experience greater indoor heat stress because thermal comfort depends not only on air temperature, but also on radiant heat from hot ceilings, walls, windows, and floors. Building-science evidence indicates that, under identical conditions, a 1950s-era home may feel substantially warmer than a newer home because poorly insulated surfaces and single-glazed windows increase mean radiant and operative temperatures. Additional housing-performance guidance also notes that older homes often struggle to stay cool because of poor insulation, inefficient airflow, leaky ductwork, single-pane windows, and aging HVAC systems. Because direct county-level data on insulation quality, indoor temperature, air-conditioning access, HVAC efficiency, energy burden, and building condition are not consistently available across all U.S. counties, housing age provides a practical proxy for housing-related heat vulnerability. However, housing age alone may not capture newer low-income housing with inadequate cooling access, high cooling costs, or substandard construction quality. Therefore, Old Housing is interpreted as a partial indicator of housing-related vulnerability rather than a direct measure of indoor heat exposure. *[Supporting sources: University of Arizona/The Conversation: https://news.arizona.edu/employee-news/new-conversation-why-older-homes-feel-warmer-thermostat-suggests-and-what-do-about-it; https://www.shalom2u.com/older-homes-struggle-to-stay-cool/.]*

- Food Access Issue (% population living in low-access census tracts): Calculated using data from the USDA Food Access Research Atlas (2021 release). Low-access tracts were defined as census tracts where at least 500 people or 33% of the

population reside more than 1 mile (urban) or 10 miles (rural) from the nearest supermarket. County-level percentages were computed as the sum of low-access tract populations divided by total county population.

- Uninsured Population (% without health insurance): The available data from ACS Table B27001 ("Health Insurance Coverage Status by Sex by Age") reports the number of insured individuals across all age and sex categories. To derive the uninsured population, the total number of insured individuals was subtracted from the total population for whom insurance status was reported. This difference was then divided by the total population and multiplied by 100 to express the uninsured population as a percentage.

- Medical Dependency (% using durable medical equipment): Data on individuals dependent on electricity-powered durable medical equipment (e.g., ventilators, oxygen concentrators) were obtained directly from the U.S. Department of Health and Human Services (HHS) emPOWER Program, current as of April 2024. Values reflect the proportion of the county's population registered in the emPOWER dataset.

- Delayed Emergency Room (ER) Access

  Two metrics were computed to assess timely emergency medical care access:

  - Minimum Distance to Nearest ER (in miles): Calculated as the straight-line distance between each county's population-weighted centroid and the nearest emergency facility using facility coordinates from the CMS Hospital General Information dataset (April 2025 release) and county

centroid coordinates derived from the Missouri Census Data Center's Geocorr tool.

- % Population Living Beyond 10 Miles from Nearest ER: Derived by overlaying ZIP Code Tabulation Area (ZCTA)-level ER distances onto county boundaries, and calculating the share of each county's population residing in ZCTAs where the minimum distance exceeded 10 miles. This method parallels the approach used in a national hospital access analysis(1).

These socioeconomic variables were selected because they capture different aspect of local vulnerability that are relevant for understanding disparities in exposure to compound drought and heatwave (CDHW) events. Economic indicators such as poverty and agricultural employment reflect financial constraints and occupational exposure, which may increase susceptibility to heat and drought impacts. Demographic factors, including elderly populations and low educational attainment, can heighten health risks and limit adaptive capacity. Infrastructure-related measures, such as old housing and food access, influence physical exposure and resilience during extreme events, while health and medical vulnerabilities like insurance coverage, medical dependency, and emergency care access directly affect the ability to cope with heat-related health crises. To facilitate analysis and interpretation, these variables were grouped into four thematic categories—economic vulnerability, demographic sensitivity, infrastructure and built environment vulnerability, and health and medical vulnerability, based on the similarity of their underlying traits and potential roles in shaping CDHW risk.

A.2 Spatial Interpolation by Voronoi Tessellation

Voronoi tessellation is a method used to divide a space into regions based on the distance to a set of specific points, called seeds or sites. Each region, known as a Voronoi cell, contains all locations that are closer to its seed point than to any other seed point. This technique is widely used for spatial interpolation because it allows information from known locations to be extended into nearby areas where data might be missing. As shown in Supplementary Figure S1, the process of creating a Voronoi tessellation involves several steps. First, the seed points are plotted (top-left panel). Then, lines are drawn connecting nearby seed points (top-right). Next, perpendicular bisectors are calculated between these lines to define boundaries (bottom-left). Finally, these boundaries form the Voronoi polygons (bottom-right), where each polygon encloses the area closest to its corresponding seed point. In this study, we used Voronoi tessellation to fill in missing drought and heatwave data at the county level. Specifically, the centroids of counties with known drought or heatwave status were used as seed points. We constructed a Voronoi diagram using the *scipy.spatial.Voronoi* function in Python. For counties without data, we determined in which Voronoi polygon their centroid fell. These counties were then assigned the drought and heatwave status of the nearest neighboring county corresponding to that polygon. This approach ensured that the spatial datasets for drought and heatwave conditions were complete across all counties.

A.3 Spatial Clusters of Occupational Engagement and Population Change

To identify clusters of counties sharing similar occupational characteristics and patterns of population change, we performed a spatial connectivity analysis based on county

boundaries. Occupational engagement was classified into binary categories indicating whether a county was predominantly outdoor-oriented (e.g., farming, mining, recreation) or indoor-oriented (e.g., manufacturing, government). For population change, counties were marked as experiencing significant demographic shifts if they fell within the upper quartile of absolute percentage change, capturing both substantial increases and substantial declines in population between 2010 and 2021, based on U.S. Census data. The analysis was implemented using the Python libraries GeoPandas and NetworkX. County geometries were imported from shapefiles via GeoPandas. For each group of interest (e.g., counties with large population changes), a spatial adjacency graph was constructed in NetworkX, where nodes represented individual counties and edges connected counties sharing a boundary (using the Queen contiguity criterion). Connected components within the graph were identified as clusters of contiguous counties exhibiting the same binary attribute. Clusters composed of a single county were excluded to focus on spatially coherent groupings of practical significance. Cluster identifiers were then merged back into the geographic data for subsequent mapping and analysis. This approach allowed us to delineate regions where counties with similar occupational structures or significant demographic shifts are spatially connected.

A.4 Specification of Penalized Complexity Priors and INLA Hyperparameters

To model spatially varying associations between compound drought and heatwave (CDHW) occurrences and socioeconomic factors, we implemented a Spatially Varying Coefficient (SVC) model in a Bayesian framework using Integrated Nested Laplace Approximation (INLA). The spatial structure of each coefficient field was modeled with

an intrinsic conditional autoregressive (ICAR) prior, also known as the Besag prior, which captures local spatial dependence by assuming neighboring regions have similar values (2, 3).

Let
$$f_k = [f_k(s_1), \ldots \ldots, f_k(s_n)]'$$
denote the vector of spatially varying coefficients for covariate k across n counties. The ICAR prior for f_k is specified via a precision matrix Q, constructed from the adjacency relationships of counties. The entries of Q are defined as follows:

$$Q_{ii} = \begin{cases} \sum_{j \neq i} W_{ij}\,, & if\ i = j \\ -W_{ij}, & if\ i \neq j \end{cases} \qquad [1]$$

where W_{ij}=1 if counties *i* and *j* are neighbors under the Queen contiguity rule, and W_{ij}=0 otherwise. Thus, the diagonal term Q_{ii} equals the number of neighboring counties for county *i*, enforcing spatial smoothing by linking each region to its immediate neighbors.

To prevent overfitting and ensure interpretable spatial smoothness, we adopted Penalized Complexity (PC) priors for the hyperparameters governing the marginal variance of each spatial field (4). The PC prior penalizes deviation from a simpler base model (a spatial field with zero variance) unless the data provide strong evidence for spatial heterogeneity.

Let σ denote the marginal standard deviation of a spatial random field. The PC prior is formulated so that:

$$\Pr(\sigma > U) = \alpha \qquad [2]$$

where U is a user-defined upper threshold and α is a small tail probability, typically chosen as 0.01. This formulation implies that there is only an α chance that σ exceeds U,

discouraging large spatial variability unless clearly supported by the data. The rate parameter λ of the corresponding exponential prior on σ is computed as:

$$\lambda = -\frac{log(\alpha)}{\mathrm{U}} \qquad [3]$$

yielding the prior density:

$$\pi(\sigma) = \lambda exp(-\lambda\sigma) \qquad [4]$$

If the precision $\tau=1/\sigma^2$ is modeled directly instead of σ, the PC prior can alternatively be expressed as a Gamma distribution, though the exponential form on σ is often preferred for clarity and is implemented directly in INLA(5, 6).

To guide the choice of U, we computed a reference standard deviation σ_{ref}, defined as the geometric mean of the marginal standard deviations across all spatial units:

$$\sigma_{ref} = exp\left(\frac{1}{n}\sum_{i=1}^{n} log(\sigma_i)\right) \qquad [5]$$

where σ_i denotes the marginal standard deviation for county *i*. In our analyses, U was typically set between 0.5 and 2 times σ_{ref}, with $\alpha=0.01$, to balance model flexibility with a conservative approach to spatial complexity. To assess sensitivity to the PC prior specification, we examined alternative upper standard-deviation thresholds (U = 0.5, 1, 2, 5), while keeping (alpha = 0.01). Smaller values of (U) impose stronger shrinkage toward the base model with limited spatial variation, whereas larger values allow greater spatial flexibility in the coefficient fields. The corresponding PC prior (b) values were 0.01288, 0.05153, 0.20612, and 1.28825, respectively. This comparison was used to document how different assumptions about spatial smoothness may affect the SVC prior structure and to support the selected prior range as a balance between spatial flexibility and regularization.

The PC prior specification was applied separately to the spatial coefficient field for each of the nine socioeconomic predictors. This allowed each predictor–CDHW association to vary smoothly across counties while controlling excessive spatial complexity. By imposing this regularization, we ensured stable estimation while preserving the capacity to detect meaningful spatial heterogeneity in the relationships between CDHW occurrence and socioeconomic vulnerability. All prior specifications and hyperparameter tuning were implemented using the R-INLA package, which offers direct support for PC priors in latent Gaussian models (7, 8).

A.5 Estimation of Moran's I for Spatial Clustering of Socioeconomic Factors

To assess the degree to which socioeconomic conditions exhibit spatial clustering across the United States, we computed Moran's I for each socioeconomic variable separately within nine predefined regions. This analysis was based on county-level data for the year 2020. Counties with missing values for a given variable were removed prior to computation. For each region, we constructed a spatial weights matrix W using first-order queen contiguity, where $w_{ij} = 1$ if counties i and j share a boundary point and 0 otherwise. The weights were row-standardized so that each row sums to one. Let x_i denote the value of a socioeconomic variable in county i, and let $\bar{x}$ be the regional mean. Moran's I for the region was then calculated as:

$$I = \frac{N}{\sum_i \sum_j w_{ij}} \frac{\sum_i \sum_j w_{ij} (x_i - \bar{x})(x_j - \bar{x})}{\sum_i (x_i - \bar{x})^2} \quad [6]$$

where N is the number of counties in the region. Positive values of I indicate that similar values tend to occur near one another (spatial clustering), values near zero suggest no meaningful spatial pattern, and negative values reflect spatial dispersion. Higher Moran's

I values correspond to more spatially concentrated patterns of the socioeconomic variable within the region, while lower values indicate more diffuse or scattered spatial distributions. These estimates were used to characterize the spatial organization of vulnerability across regions and are displayed in Supplementary Fig. S-8.

To assess the uncertainty of the regional Moran's I estimates, we applied a bootstrap resampling approach. For each socioeconomic variable and region, counties were resampled with replacement within the same region, and Moran's I was recalculated for each bootstrap sample using the same queen contiguity spatial weights and row-standardized weighting approach. This procedure was repeated 100 times for each region–variable combination. The bootstrap standard error was calculated as the standard deviation of the bootstrap Moran's I values. In Supplementary Fig. S-8, bars show the observed Moran's I values, and error bars represent the bootstrap standard error. This uncertainty estimate was used to evaluate the stability of the regional spatial clustering patterns under county-level resampling.

Supplementary Fig. S-8 shows that most regional Moran's I estimates have relatively small bootstrap standard errors, indicating that the estimated spatial clustering patterns are generally stable under county-level resampling. Some indicators show larger uncertainty in specific regions. For example, delayed ER access shows comparatively larger uncertainty in the Northeast, while food access issue also shows wider error bars in selected regions. Nevertheless, the main regional patterns remain clear, with medical dependency, old housing, uninsured population, and poor population generally showing stronger spatial clustering than delayed ER access.

A.6 Categorization of Pairwise and Triplet Socioeconomic Vulnerability Groups

For each county, we categorized the value of every socioeconomic variable into three levels (Low, Moderate, and High) using the 33rd and 66th percentiles of that variable's distribution across all counties in the continental United States as thresholds. Counties with values below or equal to the 33rd percentile were assigned to the Low category, those between the 33rd and 66th percentiles to the Moderate category, and those above the 66th percentile to the High category. This classification ensured balanced group sizes while capturing important differences in socioeconomic conditions. In the pairwise analysis, we examined all unique combinations of two socioeconomic variables, resulting in 36 distinct pairs. For each pair, the three-level categorization led to nine possible joint categories, such as Low–Low, Low–Moderate, or High–High. The Low–Moderate–High classification was used to create an interpretable vulnerability-regime framework for national-scale comparison, rather than to estimate a continuous effect of each socioeconomic indicator. Because the objective of this analysis is to examine how spatial heterogeneity in CDHW occurrence changes when multiple socioeconomic conditions overlap, tercile grouping provides a consistent way to compare counties with relatively low, moderate, and high levels of vulnerability across all indicators and pairwise combinations.

We extended this approach to a triplet analysis, considering every unique combination of three variables. This produced 84 different triplets. For each triplet, the categorization into Low, Moderate, and High for all three variables resulted in 27 possible joint categories, such as Low–Low–Low, Low–Moderate–High, and up to High–High–High. Within each pairwise or triplet grouping, we calculated the Gini coefficient to assess spatial heterogeneity in CDHW occurrence. This metric reflects how evenly or unevenly severe

CDHW days are distributed across counties within each category. Higher Gini values indicate that a small number of counties bear a disproportionate share of CDHW occurrence, highlighting areas of concentrated risk.

To assess uncertainty in the pairwise Gini coefficient estimates, we applied a bootstrap resampling approach. For each two-variable socioeconomic combination and paired vulnerability category, counties were resampled with replacement within the same category. For example, for the Old Housing–Medical Dependency pair, High–Low indicates counties with high Old Housing vulnerability and low Medical Dependency vulnerability. The Gini coefficient was then recalculated for each bootstrap sample. This procedure was repeated 100 times for each category, and the bootstrap standard error was calculated as the standard deviation of the bootstrap Gini coefficients. These uncertainty estimates were used to evaluate the stability of the observed spatial heterogeneity patterns across paired socioeconomic vulnerability categories.

Figure S-13 shows uncertainty in the pairwise Gini coefficient estimates. Most paired socioeconomic categories show relatively small bootstrap standard errors, indicating that the estimated spatial heterogeneity patterns are generally stable under county-level resampling. Wider error bars are observed for a limited number of categories, including Poverty & Old Housing in the Moderate–High category, Elderly & Old Housing in the Low–High category, Low Education & Uninsured in the Moderate–Moderate category, and Old Housing & Medical Dependency in the High–Low category. These cases indicate greater bootstrap variability and should be interpreted with caution. Despite this uncertainty in a small number of categories, the main differences among paired vulnerability groups remain clear.

A.7 Statistical Associations Between Population Change and CDHW Occurrences

To evaluate whether severe CDHW occurrences is associated with long-term shifts in regional population dynamics, we formulated a testable hypothesis using a linear regression framework. Regional population change was measured as the percent change from 2010 to 2020, and severe CDHW occurrences was summarized using mean CDHW duration for the period 2011–2020, referenced against duration changes observed during 2000–2010. Population change in each region was modeled as a function of CDHW occurrences and key socioeconomic conditions:

$$\Delta Pop_r = \alpha + \beta_1 CDHW_r + \beta_2 Poverty_r + \beta_3 AgEmployment_r + \varepsilon_r \qquad [7]$$

Here, $\Delta \mathrm{Pop}_r$ denotes the percent population change in region r from 2010 to 2020, and CDHW_r represents the average severe CDHW occurrence during 2011–2020. Socioeconomic covariates were included to reflect structural characteristics that shape population dynamics independently of climate conditions. Potential multicollinearity among regression predictors was assessed using variance inflation factors (VIFs). VIF quantifies the extent to which the variance of an estimated regression coefficient is inflated because of linear dependence among predictors in the model. For each regional regression, VIFs were calculated using the variance_inflation_factor function from the statsmodels.stats.outliers_influence module in Python. The VIF values reported in Supplementary Table S3 range from approximately 1 to 2.8, indicating low to moderate multicollinearity among predictors. Because all VIF values were below the commonly used threshold of 5, no serious multicollinearity was detected in the regional regression models.

We hypothesize that higher severe CDHW occurrence is associated with slower population growth or population decline.

- H_0 (null): $\beta_1 = 0$ severe CDHW occurrence is not systematically associated with population change.
- H_1 (alternative): $\beta_1 < 0$ regions with greater severe CDHW occurrence experience slower growth or population decline.

This hypothesis directly tests whether climate stress exerts a measurable influence on demographic trends, after controlling for socioeconomic conditions.

Table 2 presents the regional regression results. Significance levels are indicated by stars, where *** ($p < 0.001$), ** ($0.001 \leq p < 0.01$), * ($0.01 \leq p < 0.05$), and NS indicates no statistical significance. CDHW occurrence exhibits heterogeneous associations with population change across U.S. regions. Significant negative coefficients in the Central, East North Central, West, and West North Central regions indicate that higher CDHW occurrence is linked with population decline after adjusting for poverty and agricultural employment. By contrast, the Southeast and Northeast show significant positive associations, suggesting that population growth continues even with increased CDHW duration relative to their own historical baselines; this may reflect ongoing urban expansion and sustained in-migration rather than direct sensitivity to climate stress. In the South, Southwest, and Northwest, CDHW occurrence does not exhibit a significant effect, implying that population patterns there are shaped more strongly by socioeconomic factors than by climate stress alone. Across all regions, poverty and agricultural employment show consistent negative associations with population change, highlighting that structurally

disadvantaged or agrarian counties tend to lose population regardless of climate exposure. These contrasting regional patterns underscore that demographic responses to severe CDHW events are highly context-dependent and shaped by the interaction of climate stress with underlying socioeconomic conditions.

A.8 Sensitivity of Socioeconomic Binning Strategy

Socioeconomic indicators such as poverty rate vary continuously across space and do not exhibit clear natural breakpoints. Consequently, grouping schemes based on coarse percentile classes (e.g., terciles or quartiles) may obscure gradual socioeconomic gradients and potentially mask monotonic relationships between vulnerability and climate hazard metrics. To better resolve progressive changes in vulnerability, several recent climate–society studies have adopted fixed-interval binning combined with regression on bin medians, which reduces sensitivity to outliers while preserving large-scale socioeconomic trends (9). In the main analysis, we use fixed 5% poverty intervals to examine the relationship between poverty share and CDHW duration. This bin width provides sufficient resolution to capture gradual socioeconomic transitions while maintaining adequate sample sizes within each group. In addition, because relatively few counties exceed poverty rates of 30–40%, all values above 30% are aggregated into a single upper-tail bin to avoid unstable statistics associated with sparse sampling at extreme poverty levels. To assess the robustness of this binning choice, we conducted a sensitivity analysis using alternative fixed bin widths of 3%, 4%, and 5%, while maintaining the same upper-tail aggregation (>30%). For each binning configuration, boxplots were constructed, and linear regression was performed between bin-center poverty values and median CDHW duration, following the same procedure as in the main analysis. The results are consistent across all bin widths.

Specifically, for 3% bins (11 groups), the correlation coefficient is r = 0.65 with p = 0.032; for 4% bins (9 groups), r = 0.63 with p = 0.071; and for 5% bins (7 groups), r = 0.69 with p = 0.085. Although statistical significance weakens slightly for coarser binning due to the reduced number of bins, correlation magnitudes remain comparable and positive across all configurations, indicating a stable monotonic relationship between poverty share and CDHW duration. These results demonstrate that the inferred socioeconomic gradient in CDHW occurrence is robust to the choice of binning strategy; therefore, we retain 5% bins in the main figures as they provide an optimal balance between resolving gradual socioeconomic variability and maintaining a sufficient number of bins.

A.9 Voronoi-based spatial imputation and validation of missing county-level drought information

Some counties did not have complete drought information in the original county-level dataset. To retain these counties in the spatial analysis, missing records were filled using a nearest-neighbor spatial imputation procedure. The method was based on the assumption that counties located close to one another are more likely to share similar drought evolution than counties located farther apart. Each county was represented by the centroid of its polygon. Counties with available drought information were treated as reference counties, while counties without complete drought information were treated as missing counties. For each missing county, the centroid-to-centroid distance between that county and every reference county was calculated. The reference county with the shortest distance was selected as the donor county. Here, the donor county refers to the nearest county with observed drought information whose complete drought information was used to fill the missing county. The missing county was then assigned the full temporal drought

information of the nearest donor county. This procedure is equivalent to a Voronoi-based nearest-neighbor assignment, in which each missing county falls within the spatial influence region of the closest county with observed drought information.

To assess whether this imputation approach was reliable, we performed a leave-one-county-out validation using counties that already had observed drought information. In each validation step, one observed county was temporarily removed from the dataset and treated as if it were missing. The same nearest-neighbor imputation procedure was then applied to reconstruct the withheld county using the nearest remaining county with available drought information. In this validation, the withheld county was the target county, and the nearest remaining observed county was the donor county. The reconstructed drought information from the donor county was then compared with the original observed drought information of the withheld target county. The validation was performed at the temporal record level. For each withheld county, the observed and imputed drought categories were compared date by date using only dates available in both the target and donor county records. A correct match was counted when the observed drought category in the withheld target county was the same as the imputed category from the donor county on the same date. A mismatch was counted when the two categories differed. County-level agreement was calculated as the percentage of matching dates for each withheld county. Overall agreement was calculated by pooling all county-date comparisons from the full leave-one-county-out validation experiment. The validation results are shown in Fig. S-14.

Figure S-14a shows the distribution of county-level temporal agreement. Each value represents the percentage of dates for which the imputed drought category matched the observed category for a withheld county. Most counties had high agreement, with many

validation scores falling between approximately 80% and 95%, indicating that the nearest-neighbor donor county generally reproduced the temporal drought information of the withheld county. Figure S-14b shows the relationship between donor-county distance and validation agreement. The x-axis represents the centroid-to-centroid distance between the withheld target county and its nearest donor county. The y-axis represents the temporal agreement between the observed drought information of the target county and the imputed drought information taken from the donor county. Most donor counties were located within approximately 30–60 km of the withheld county. Agreement was generally high for nearby donors but became more variable as donor distance increased. This pattern is consistent with the expectation that drought similarity is stronger between nearby counties and weaker when the nearest available donor county is farther away.

Figure S-14c shows the spatial distribution of county-level validation agreement. Each mapped value represents the temporal agreement obtained when that county was withheld and reconstructed from its nearest donor county. High agreement was widespread across much of the study domain, while lower agreement appeared in some spatially heterogeneous regions. These lower-agreement areas may reflect larger county sizes, local topographic differences, irrigation effects, land-cover variation, or regional climate gradients that can weaken the similarity between a target county and its nearest donor county. Figure S-14d shows the confusion matrix comparing observed and imputed drought categories across all county-date validation records. In this matrix, the observed class is the drought category originally recorded for the withheld target county on a given date, and the imputed class is the drought category assigned from the nearest donor county on the same date. Correct classifications occur along the diagonal, while off-diagonal

entries represent temporal mismatches between the target and donor county records. The drought categories include “No Drought,” “Abnormally Dry,” and “Drought.” Here, “No Drought” represents non-drought conditions, “Abnormally Dry” represents a transitional dry condition, and “Drought” represents counties classified as being under drought conditions. The validation produced an overall agreement of 87.91%, indicating that the Voronoi-based nearest-neighbor approach correctly reproduced the observed drought category for most county-date records. Class-specific agreement was 61.16% for “Abnormally Dry,” 89.78% for “Drought,” and 93.19% for “No Drought.” The higher agreement for the “Drought” and “No Drought” categories indicates that the method more reliably captures persistent drought and non-drought conditions. The lower agreement for “Abnormally Dry” is expected because this category represents a transitional condition between non-drought and drought and is therefore more spatially and temporally variable.

Supplementary Figures

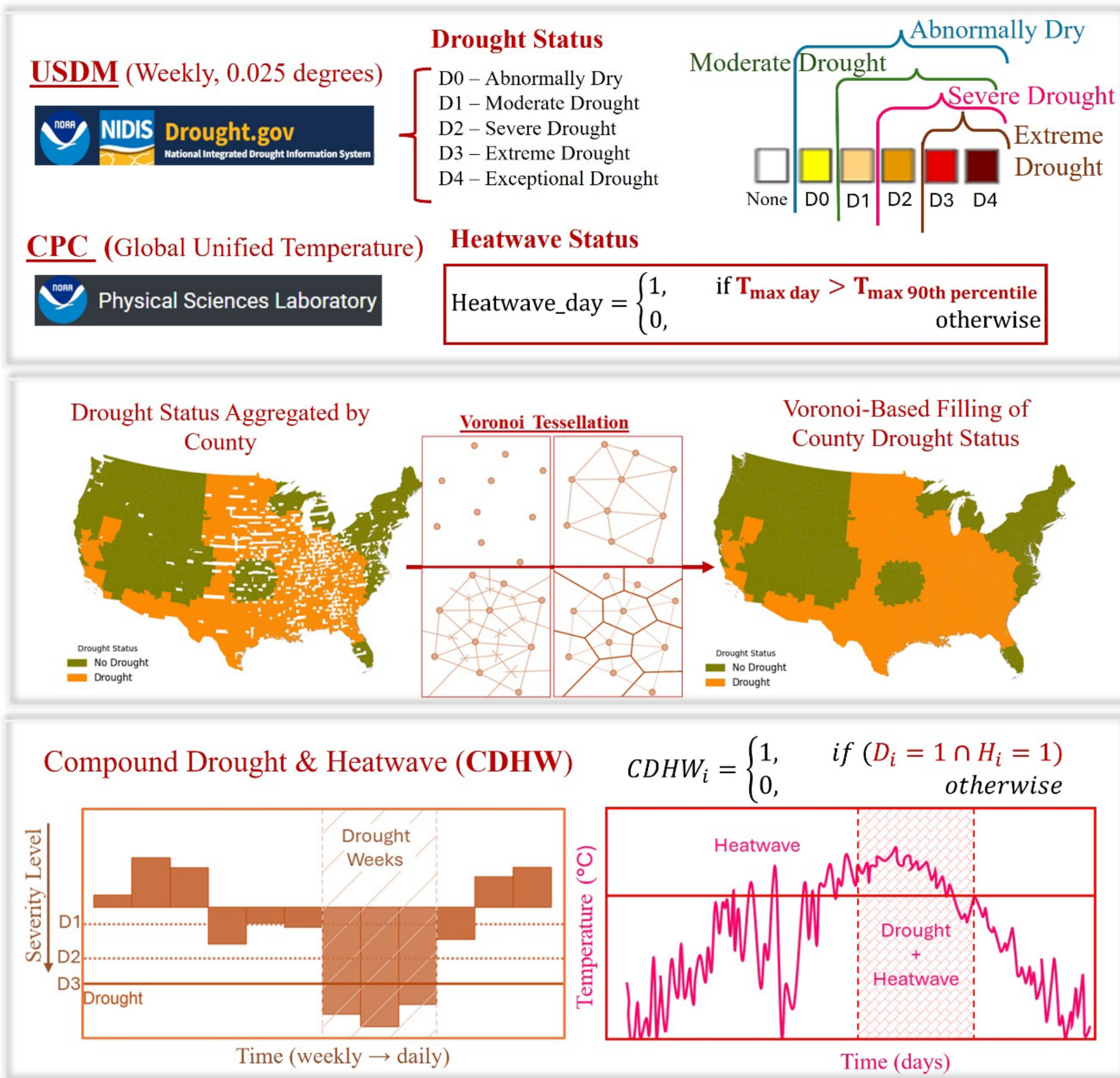

Figure S-1. Identification of Compound Drought and Heatwave Events: Schematic of the method for identifying compound drought and heatwave (CDHW) events, integrating USDM drought data and CPC temperature records. The approach includes spatial interpolation using Voronoi tessellation to fill missing county-level drought or heatwave status.

Interpretation for Figure S-1. This figure illustrates the procedure used to identify compound drought and heatwave events by combining weekly drought status from the U.S. Drought Monitor with daily heatwave status from CPC temperature records. CDHW occurrence is defined when drought and heatwave conditions overlap in the same county and time period. The Voronoi interpolation step fills missing county-level drought or heatwave information using the nearest available neighboring county, allowing a spatially complete CDHW dataset across the CONUS.

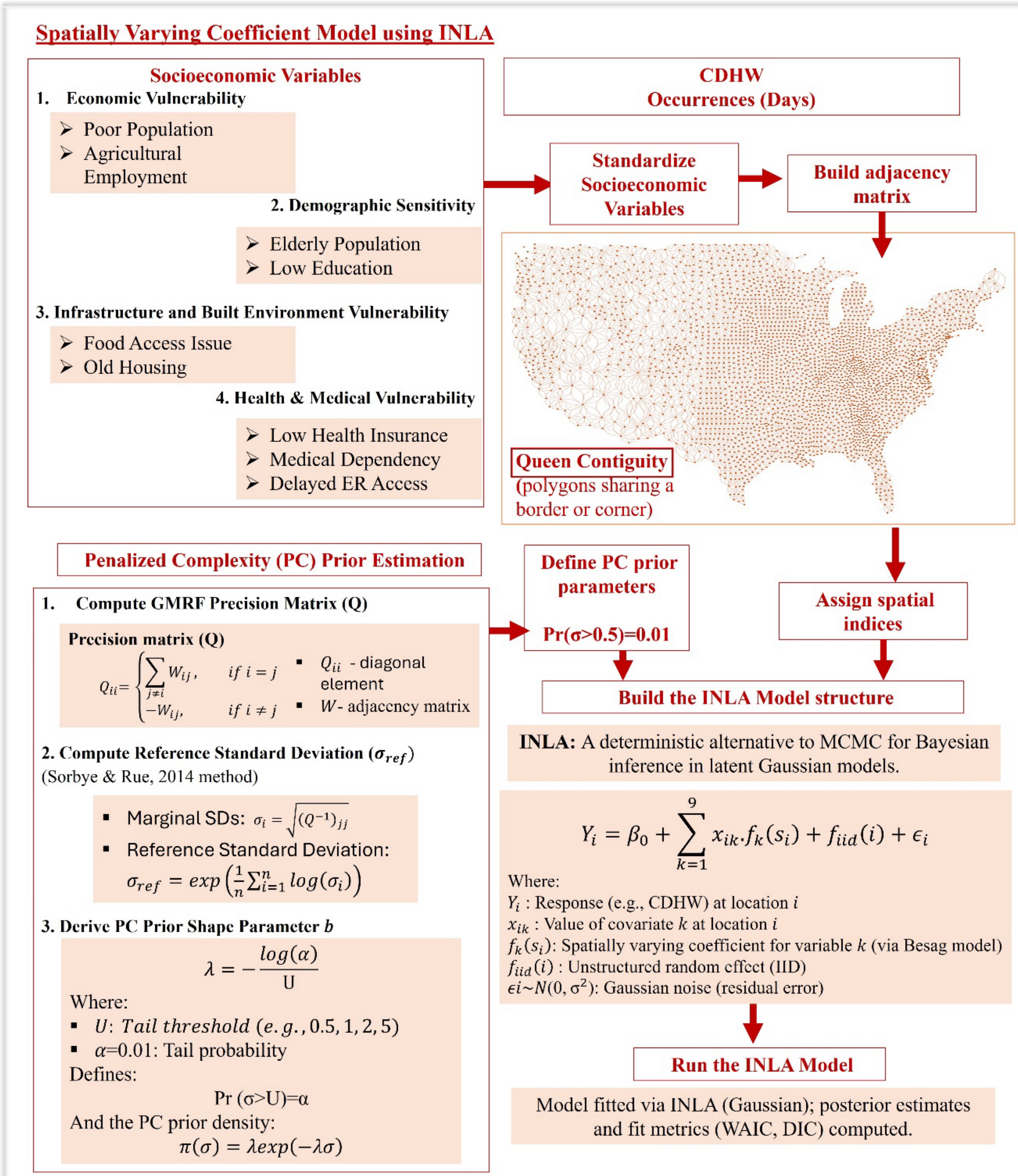

Figure S-2. Spatially varying coefficient modeling framework using INLA: Workflow outlining the steps to relate severe compound drought and heatwave (CDHW) events to socioeconomic variables across U.S. counties. The process includes building spatial adjacency matrices, defining penalized complexity priors, and fitting the model via INLA to estimate spatially varying associations between CDHW occurrence and multiple vulnerability factors.

Interpretation for Figure S-2. This figure summarizes the spatially varying coefficient modeling framework used to examine associations between severe CDHW occurrence and socioeconomic vulnerability. Socioeconomic variables are standardized, county adjacency relationships are used to represent spatial dependence, and INLA is applied to estimate geographically varying associations. This framework allows the relationship between CDHW occurrence and vulnerability indicators to vary across regions rather than assuming a single national association.

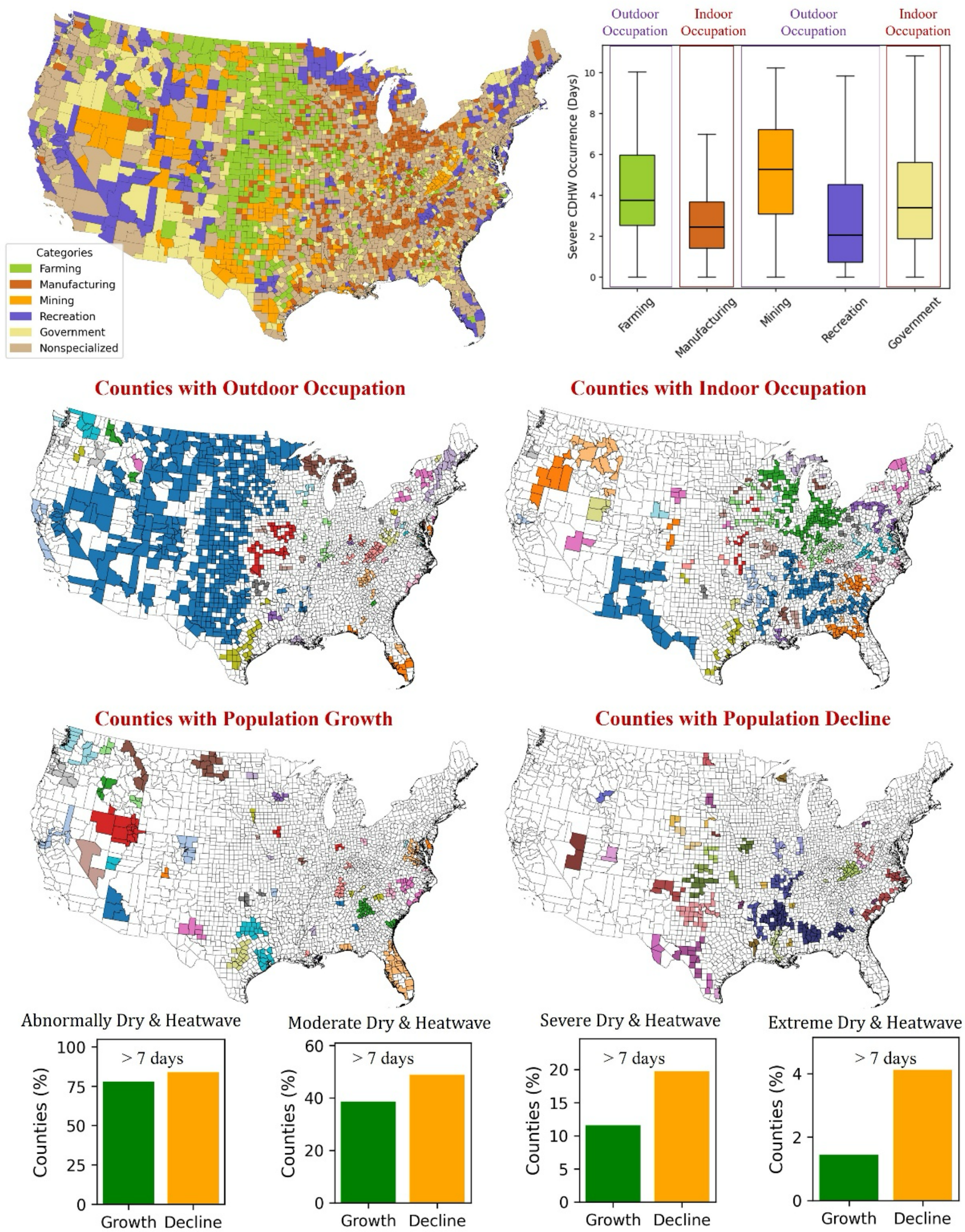

Figure S-3. Occupational and demographic patterns associated with severe CDHW occurrences across U.S. counties: The top panel shows dominant county-level occupation types and the distribution of severe CDHW days by occupation, distinguishing outdoor from indoor sectors. The lower maps illustrate spatial clusters of counties dominated by outdoor or indoor occupations, as well as clusters experiencing significant population growth or decline between the last two census periods. Both occupational and demographic clusters were identified using density-based spatial clustering techniques, revealing spatially homogeneous groups of counties.

Interpretation for Figure S-3. This figure shows how occupational structure and population change overlap with severe CDHW occurrence. Counties dominated by outdoor-oriented occupations tend to show higher CDHW exposure than many indoor-oriented counties, indicating greater potential risk for workers whose jobs require outdoor activity. The population-growth and population-decline maps show that CDHW exposure intersects with different demographic trajectories, suggesting that climate stress interacts with local economic and demographic conditions rather than producing uniform effects across all regions.

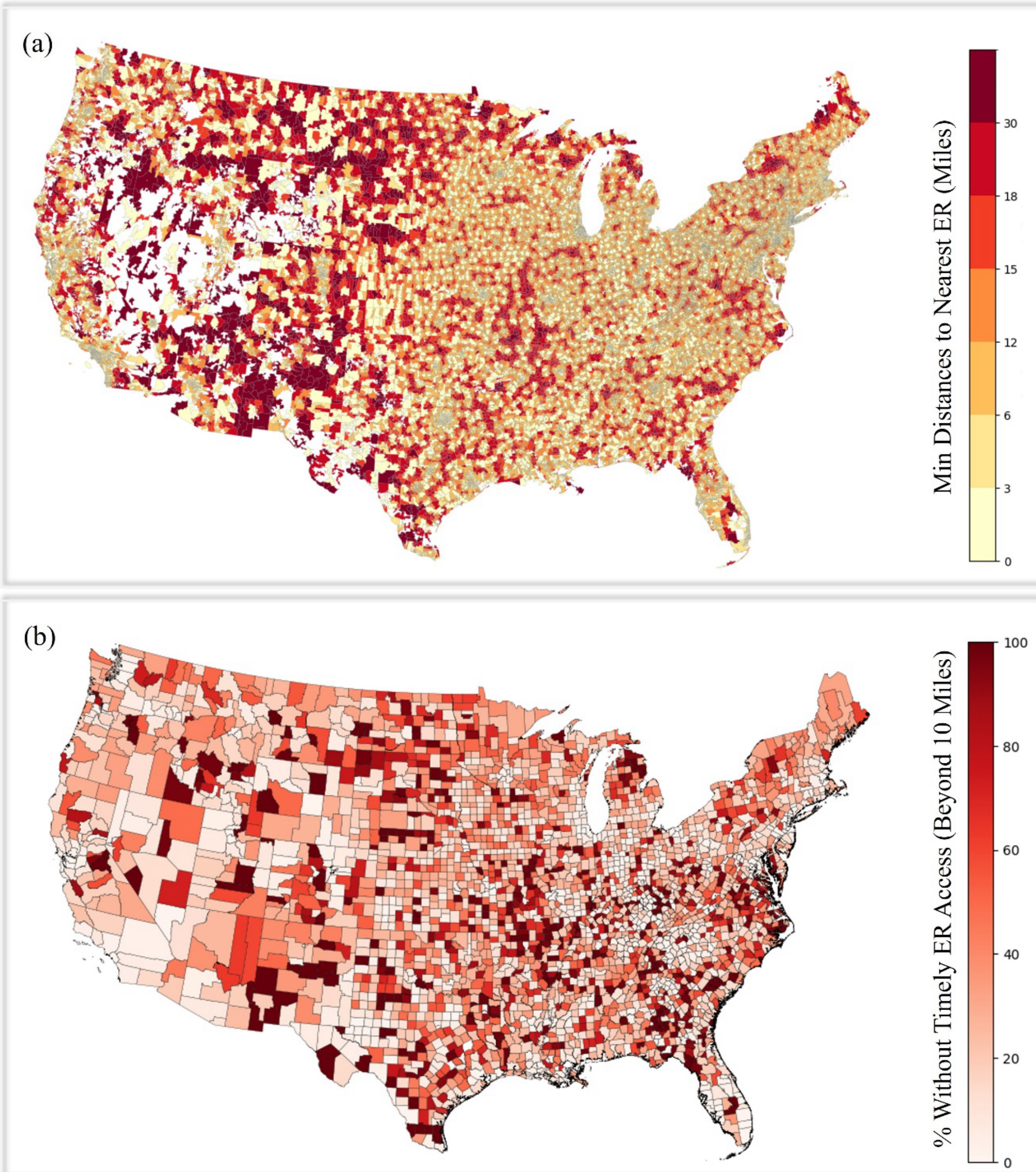

Figure S-4. Geographic patterns of emergency medical access across U.S. counties: (a) Minimum straight-line distance (in miles) from each county's population-weighted centroid to the nearest emergency room (ER) facility, aggregated from ZIP-code-level data. (b) Percentage of the county population living beyond 10 miles from the nearest ER, highlighting areas where residents may face delays in emergency care.

<u>Interpretation for Figure S-4.</u> This figure highlights geographic differences in emergency medical access, measured by distance to the nearest emergency room and the share of residents living more than 10 miles from emergency care. Delayed ER access is unevenly distributed across the United States, with many rural and interior counties facing longer

travel distances and larger populations beyond timely emergency access. During CDHW events, these spatial gaps may increase health vulnerability because rapid medical response is critical during heat-related emergencies.

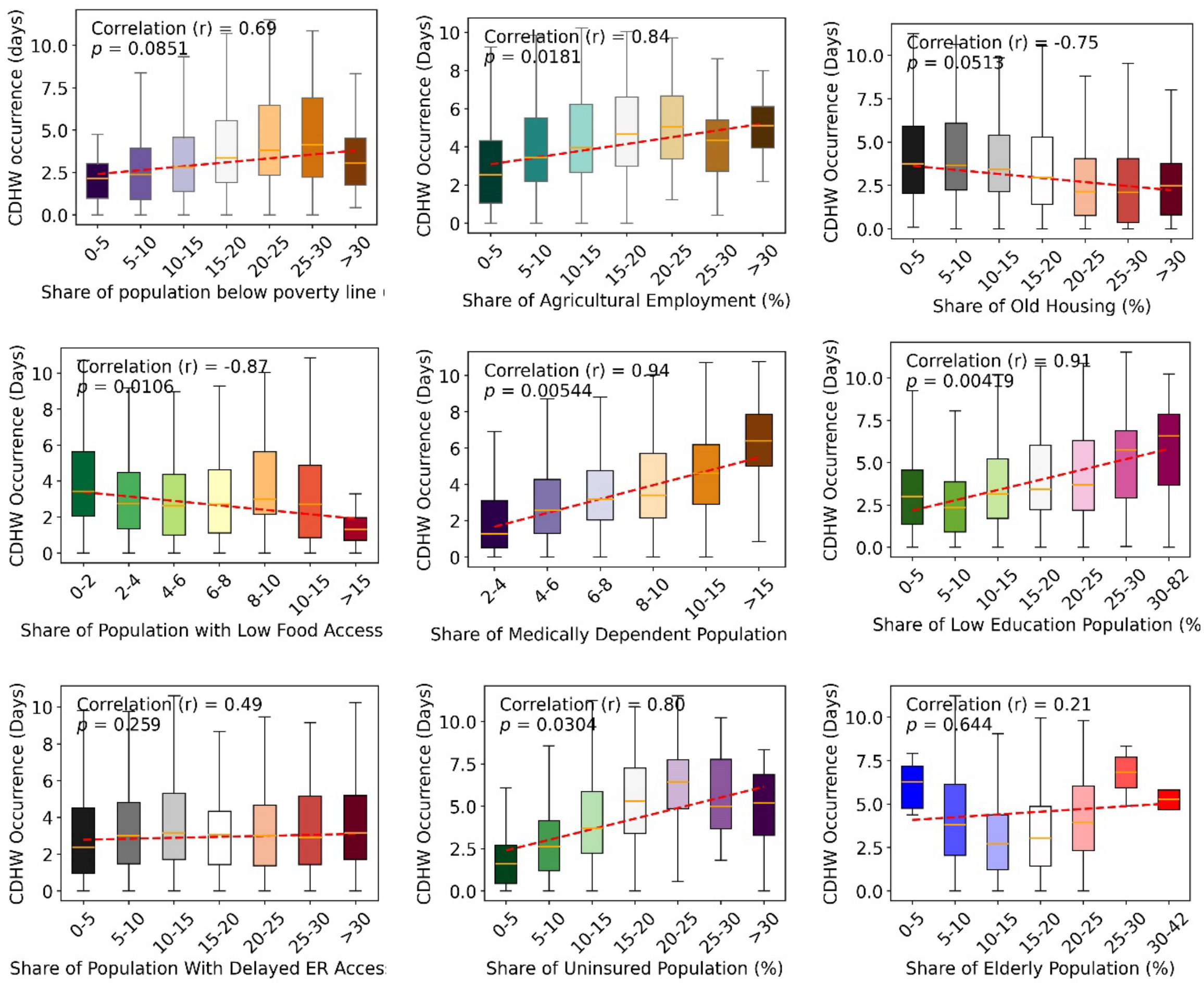

Figure S-5. Associations between severe CDHW occurrence and socioeconomic characteristics across U.S. counties. Boxplots display the distribution of severe CDHW duration (days) across binned intervals of each socioeconomic indicator, including poverty rate, agricultural employment share, old housing share, elderly population share, low education share, low food access, uninsured population share, medical dependency, and delayed emergency room (ER) access. Red dashed lines show linear trends fitted to the median severe CDHW days using the midpoint of each bin, with Pearson's *r* and two-tailed *p* values reported.

<u>Interpretation for Figure S-5.</u> This figure shows how severe CDHW duration varies across binned levels of socioeconomic vulnerability. Positive trends for several indicators suggest that counties with greater poverty, agricultural employment, low education, medical dependency, or uninsured populations often experience longer severe CDHW duration. These patterns indicate that CDHW exposure is not randomly distributed across

socioeconomic groups, but instead aligns with several dimensions of community vulnerability.

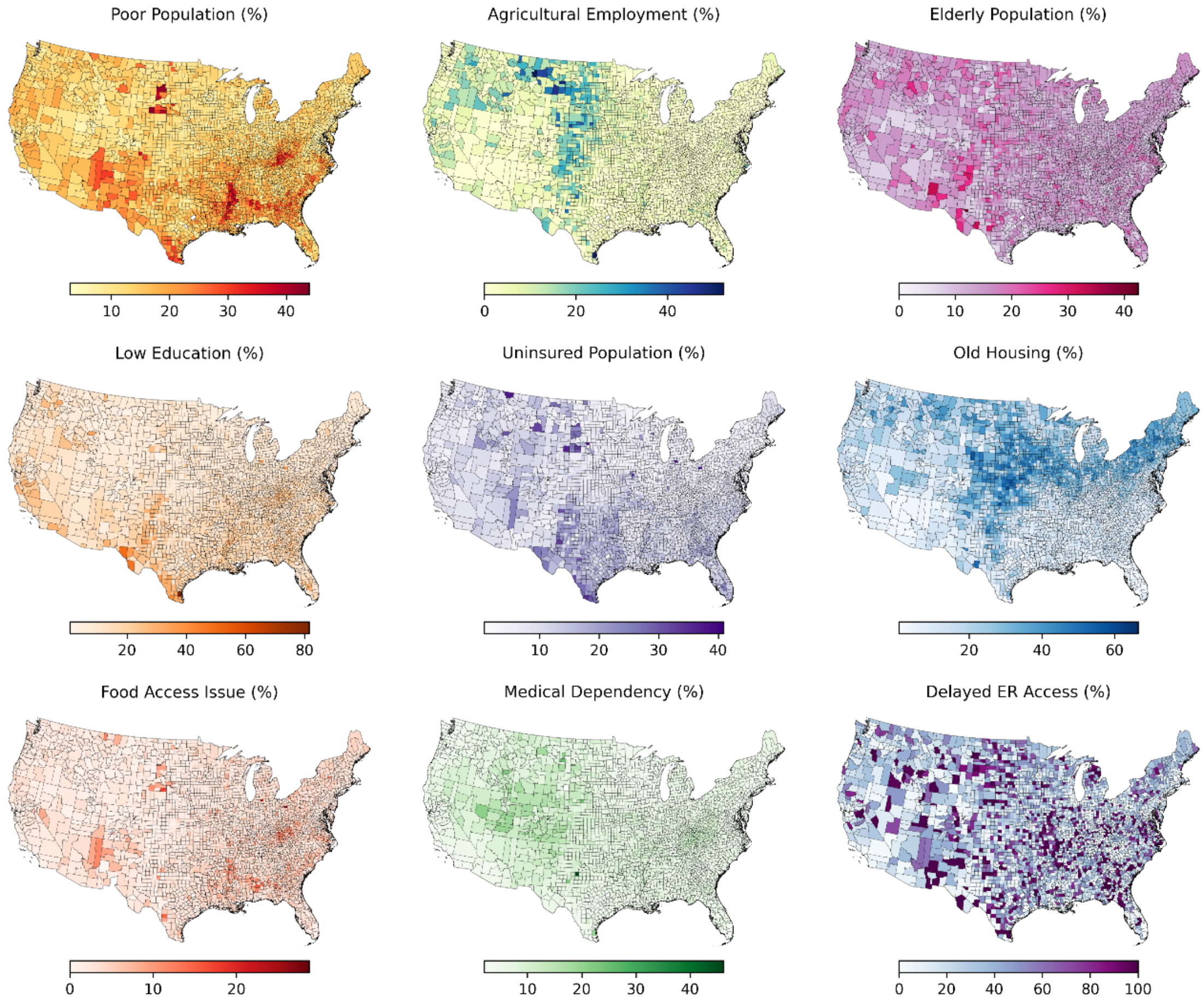

Figure S-6. Spatial distribution of socioeconomic vulnerability indicators across U.S. counties: Maps show county-level percentages for nine key socioeconomic variables examined in relation to severe CDHW occurrences: poor population, agricultural employment, elderly population, low education, uninsured population, old housing, food access issues, medical dependency, and delayed emergency room (ER) access.

<u>Interpretation for Figure S-6.</u> This figure maps the spatial distribution of the nine socioeconomic vulnerability indicators used in the analysis. Different forms of vulnerability have distinct geographic patterns, with poverty, low education, uninsured populations, old housing, food access issues, medical dependency, agricultural employment, elderly population, and delayed ER access concentrated in different regions. These spatial contrasts show that CDHW vulnerability is multidimensional and cannot be fully represented by a single socioeconomic indicator.

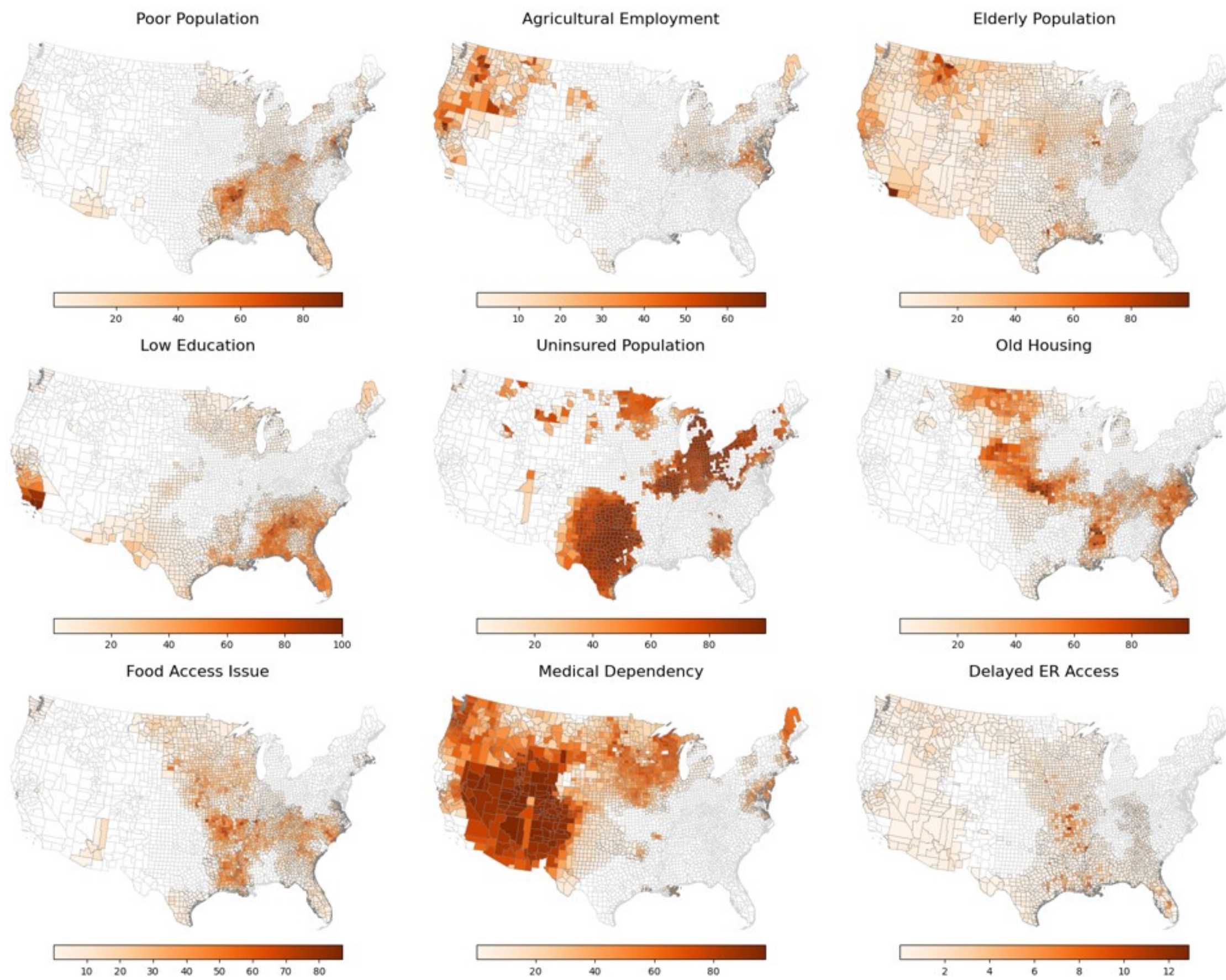

Figure S-7. County-level positive local associations between socioeconomic factors and severe CDHW days: The maps show the percentage of severe CDHW occurrences in each county that is positively associated with underlying socioeconomic conditions, revealing spatial variation in these local associations across different regions.

<u>Interpretation for Figure S-7.</u> This figure maps where severe CDHW occurrence is positively associated with each socioeconomic factor. The strength and location of these associations differ by variable, with some factors showing stronger positive associations in the South, Southwest, Great Plains, or interior regions. These results support the use of spatially varying models because the relationship between CDHW occurrence and socioeconomic vulnerability is geographically uneven.

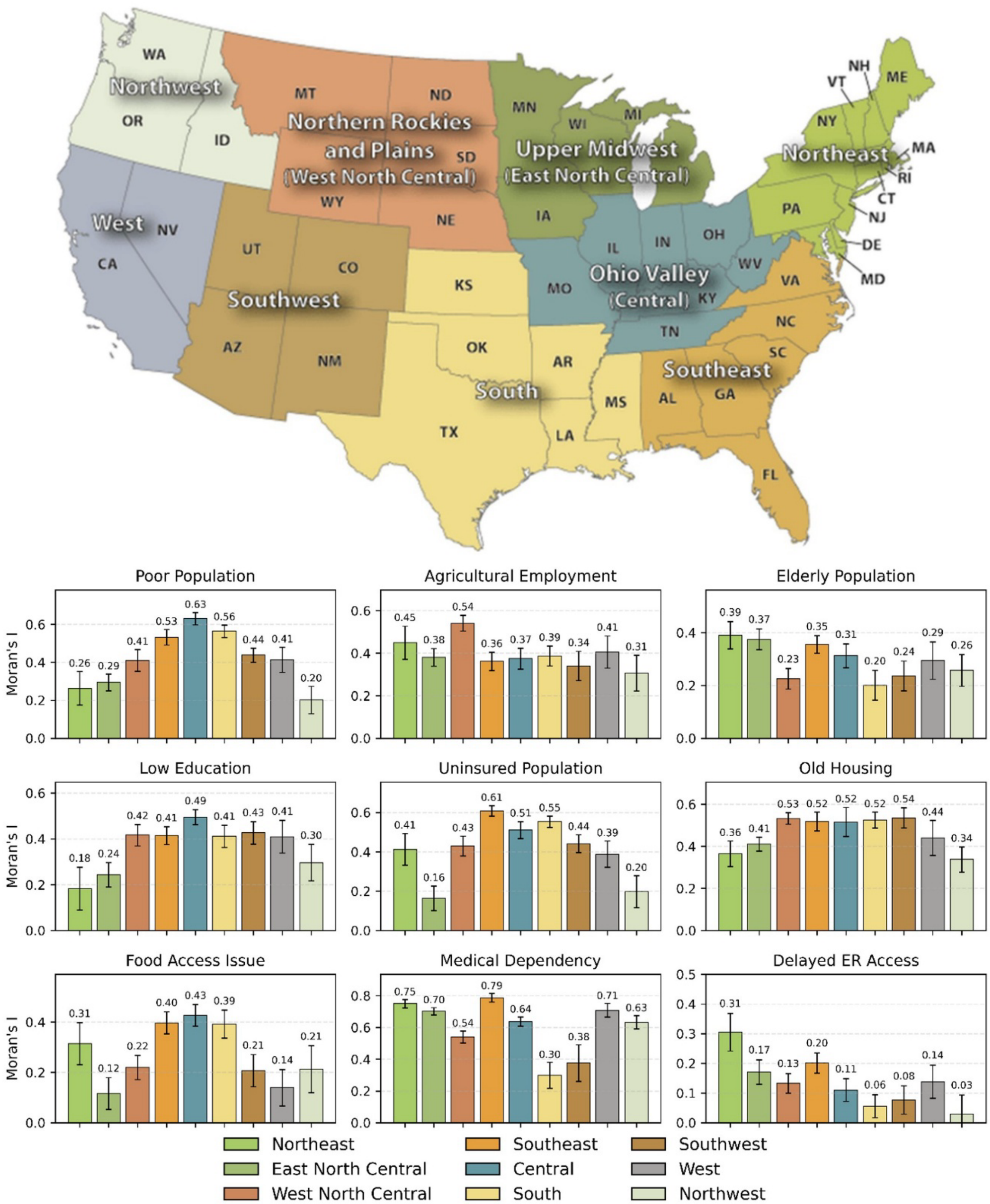

Figure S-8. Spatial Clustering of Socioeconomic Vulnerability Indicators across U.S. Regions. The map shows the U.S. climate regions used to summarize spatial patterns. Bar plots show Moran's I values for nine socioeconomic indicators within each region, measuring the degree of spatial clustering or dispersion across counties. Error bars represent bootstrap standard errors estimated from 100 county-level bootstrap resamples within each region.

Interpretation for Figure S-8. *This figure uses regional Moran's I values to show how strongly each socioeconomic vulnerability indicator clusters within U.S. climate regions. Higher Moran's I values indicate that similar county-level vulnerability values are spatially grouped rather than randomly distributed. Error bars represent bootstrap standard errors from 100 county-level resamples, providing an estimate of uncertainty around the regional Moran's I values. The results show that vulnerability is spatially structured, with some regions displaying strong clustering of poverty, uninsured populations, older housing, or medical dependency, which may contribute to contiguous areas of elevated CDHW risk.*

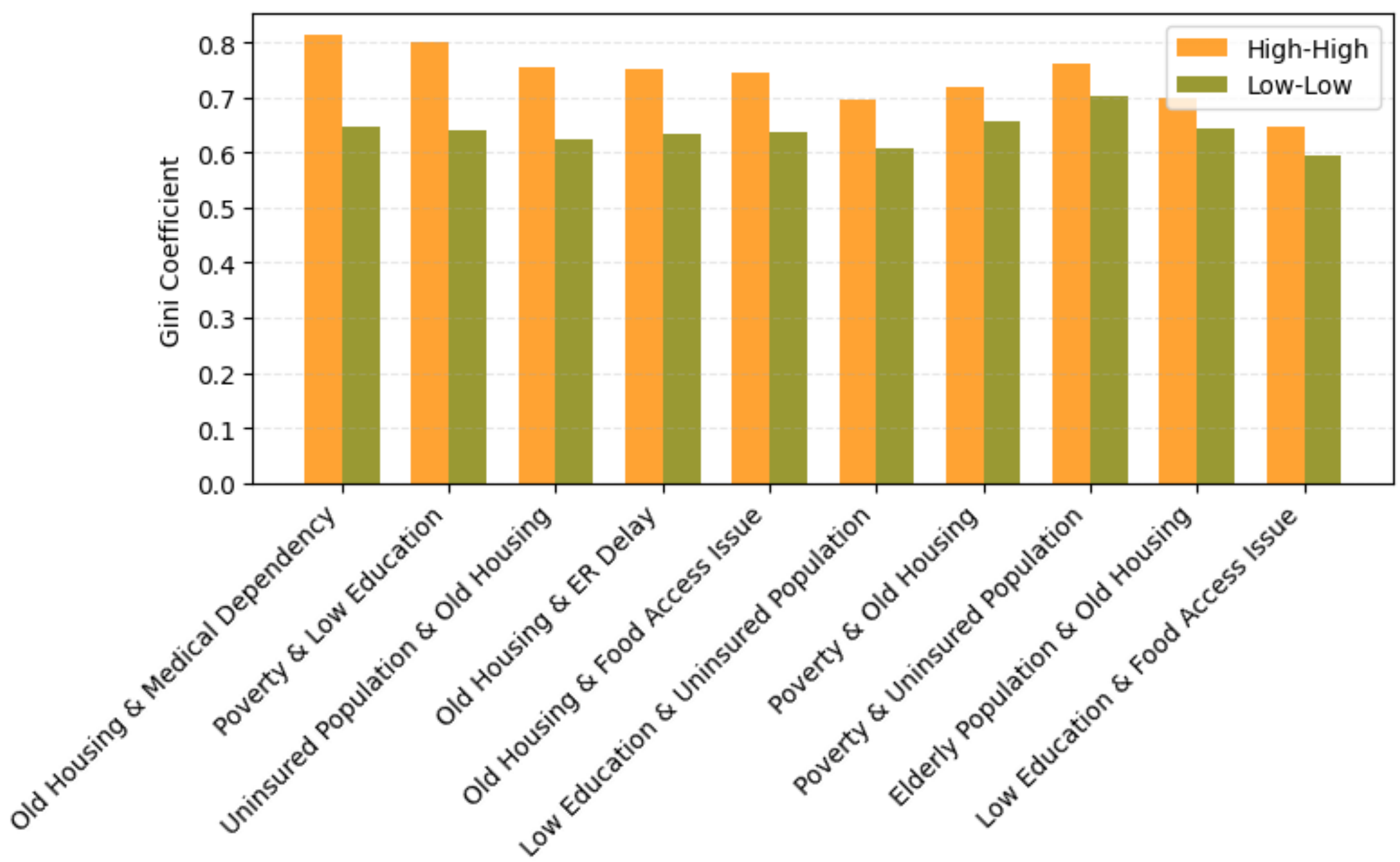

Figure S-9. Gini coefficients for paired socioeconomic vulnerabilities in relation to severe CDHW occurrences across U.S. counties. Bars show Gini coefficients quantifying **spatial heterogeneity** among counties characterized by simultaneously high levels ("High–High") or low levels ("Low–Low") of paired socioeconomic variables, highlighting differences in how combined vulnerabilities are associated with the uneven spatial distribution of severe CDHW occurrences.

Interpretation for Figure S-9. This figure compares Gini coefficients for counties with high-high and low-low paired socioeconomic vulnerabilities. Higher Gini values indicate that severe CDHW occurrences show greater spatial heterogeneity across counties within a vulnerability group. The comparison shows that overlapping vulnerabilities can concentrate CDHW occurrence in a smaller subset of counties, highlighting how compound social disadvantage may intensify spatially uneven exposure patterns.

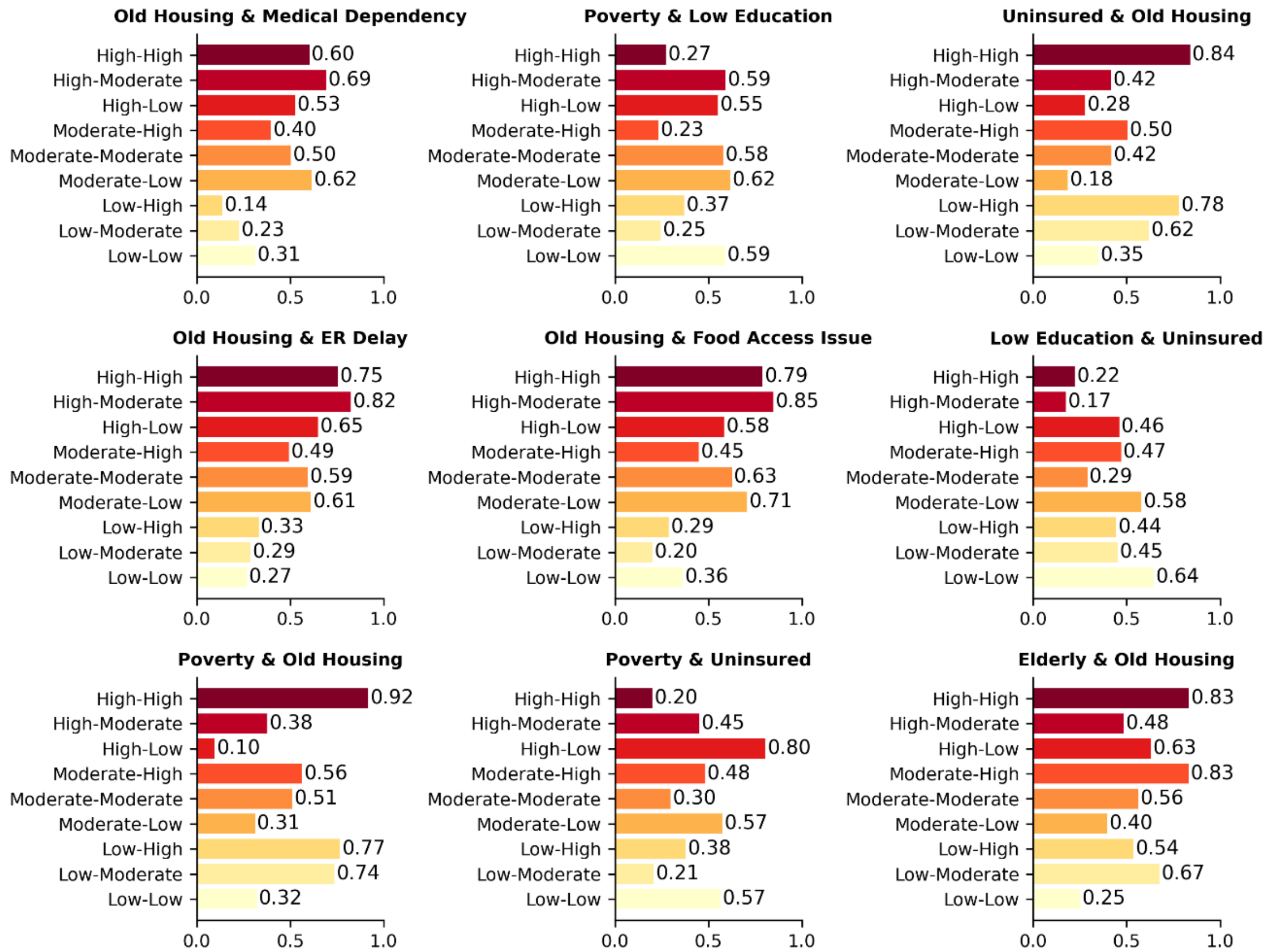

Figure S-10. Gini coefficients for combinations of paired socioeconomic vulnerabilities and severe CDHW occurrences across U.S. counties. Bars display Gini coefficients measuring spatial heterogeneity in severe CDHW days for counties grouped into combinations of high, moderate, or low levels of each pair of socioeconomic variables. Higher Gini values indicate that severe CDHW days are more unevenly distributed and concentrated within a smaller subset of counties in that vulnerability grouping.

<u>Interpretation for Figure S-10.</u> This figure shows spatial heterogeneity in severe CDHW occurrence across paired socioeconomic categories. For each socioeconomic indicator, counties were grouped into Low, Moderate, and High classes using the national 33rd and 66th percentile thresholds. Pairs of indicators were then combined to form categories such as Low–Low, Low–Moderate, High–Low, and High–High. The Gini coefficient represents the unevenness of severe CDHW-day occurrence within each paired category. A higher Gini value means that severe CDHW days are more concentrated among a smaller subset of counties in that group, rather than indicating a higher total CDHW burden or greater overall vulnerability. Thus, differences in Gini coefficients should be interpreted as differences in within-group spatial heterogeneity, not as direct rankings of total risk across vulnerability classes. This paired-indicator approach helps reveal spatially uneven occurrence patterns that may be overlooked when indicators are assessed individually.

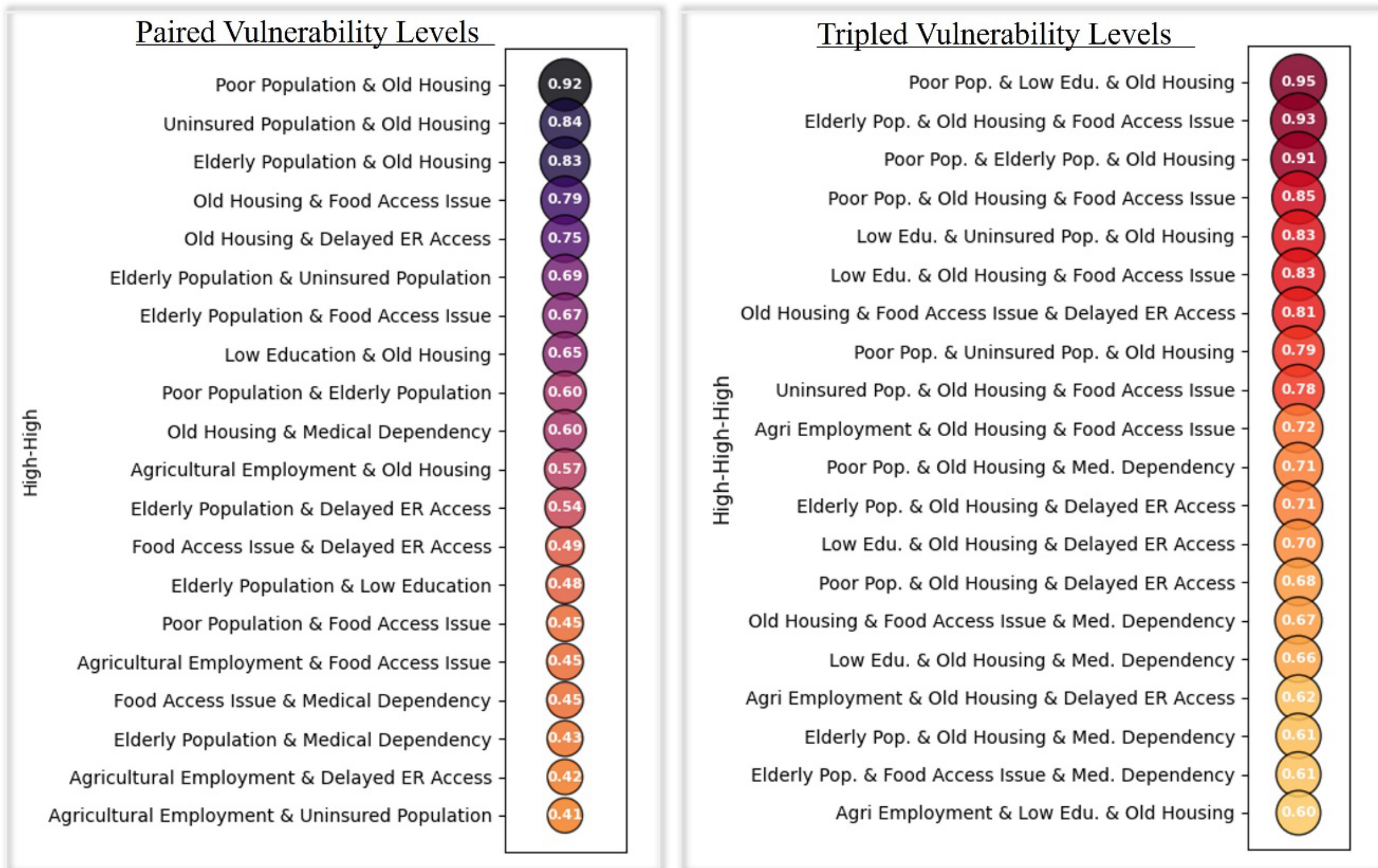

Figure S-11. Gini coefficients for paired and triplet combinations of socioeconomic vulnerabilities associated with severe CDHW occurrences across U.S. counties: The left panel shows Gini coefficients for High–High groupings of paired socioeconomic factors, indicating the degree of spatial heterogeneity in severe CDHW occurrences where two vulnerabilities coincide. The right panel presents Gini coefficients for High–High–High groupings of triplet combinations, showing how overlapping social, infrastructural, and health-related factors are associated with the uneven spatial distribution of severe CDHW occurrences.

Interpretation for Figure S-11. This figure compares spatial heterogeneity in severe CDHW occurrence for paired and triplet combinations of high socioeconomic vulnerability. The triplet combinations highlight areas where social, infrastructural, and health-related vulnerabilities overlap and where CDHW occurrence is more spatially uneven. These patterns show that CDHW burden can become more concentrated when multiple dimensions of vulnerability occur together, emphasizing the need for integrated adaptation strategies.

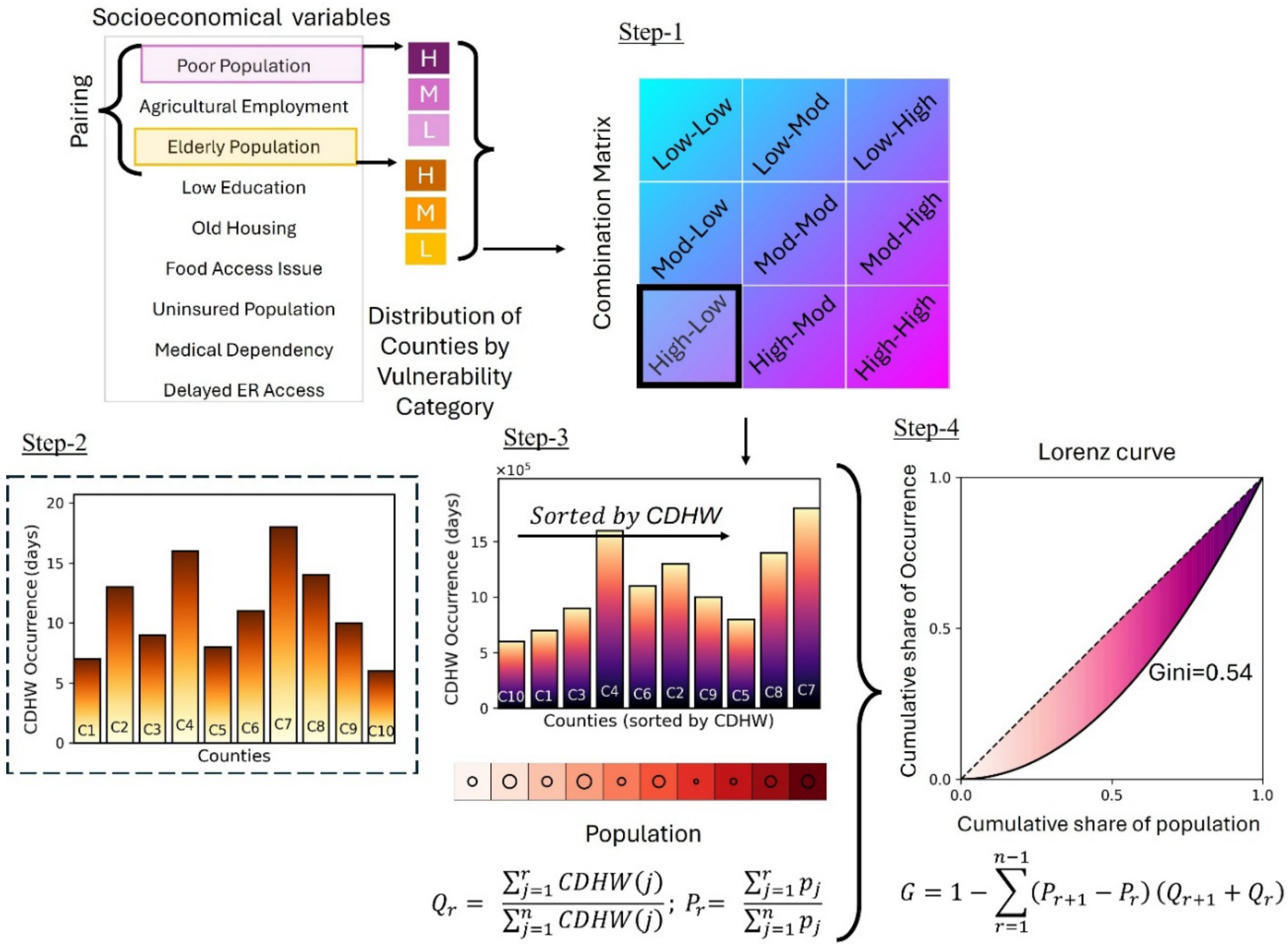

$$Q_r = \frac{\sum_{j=1}^{r} CDHW(j)}{\sum_{j=1}^{n} CDHW(j)};\; P_r = \frac{\sum_{j=1}^{r} p_j}{\sum_{j=1}^{n} p_j}$$

$$G = 1 - \sum_{r=1}^{n-1} (P_{r+1} - P_r)(Q_{r+1} + Q_r)$$

Figure S-12. Schematic workflow for calculating the Gini coefficient of CDHW occurrence across pairwise socioeconomic vulnerability groups. Step 1 classifies each socioeconomic vulnerability indicator into Low, Moderate, and High categories and combines two indicators to form a paired vulnerability matrix, such as Low–Low, Moderate–High, or High–High. Step 2 extracts the counties belonging to the selected paired vulnerability group and records their CDHW occurrence values, expressed as the average duration of severe CDHW events during the summer season. Step 3 ranks those counties from lowest to highest CDHW occurrence while retaining each county's corresponding population value; the ranked county-level values are then used to calculate cumulative population shares and cumulative CDHW occurrence shares. Step 4 uses these cumulative shares to construct the Lorenz curve and estimate the Gini coefficient. Larger deviations from the 1:1 line indicate greater concentration of CDHW occurrence within a smaller share of the population in that paired vulnerability group.

Interpretation for Figure S-12. This figure provides a step-by-step schematic of how spatial heterogeneity in CDHW occurrence was measured using Lorenz curves and Gini coefficients. Counties are grouped by socioeconomic vulnerability levels, CDHW occurrences are sorted, and the Lorenz curve and Gini coefficient summarize how unevenly CDHW days are distributed across the population. This clarification is important because the Gini coefficient is used here to measure spatial heterogeneity in CDHW occurrence across vulnerability groups, not monetary damages or realized economic losses.

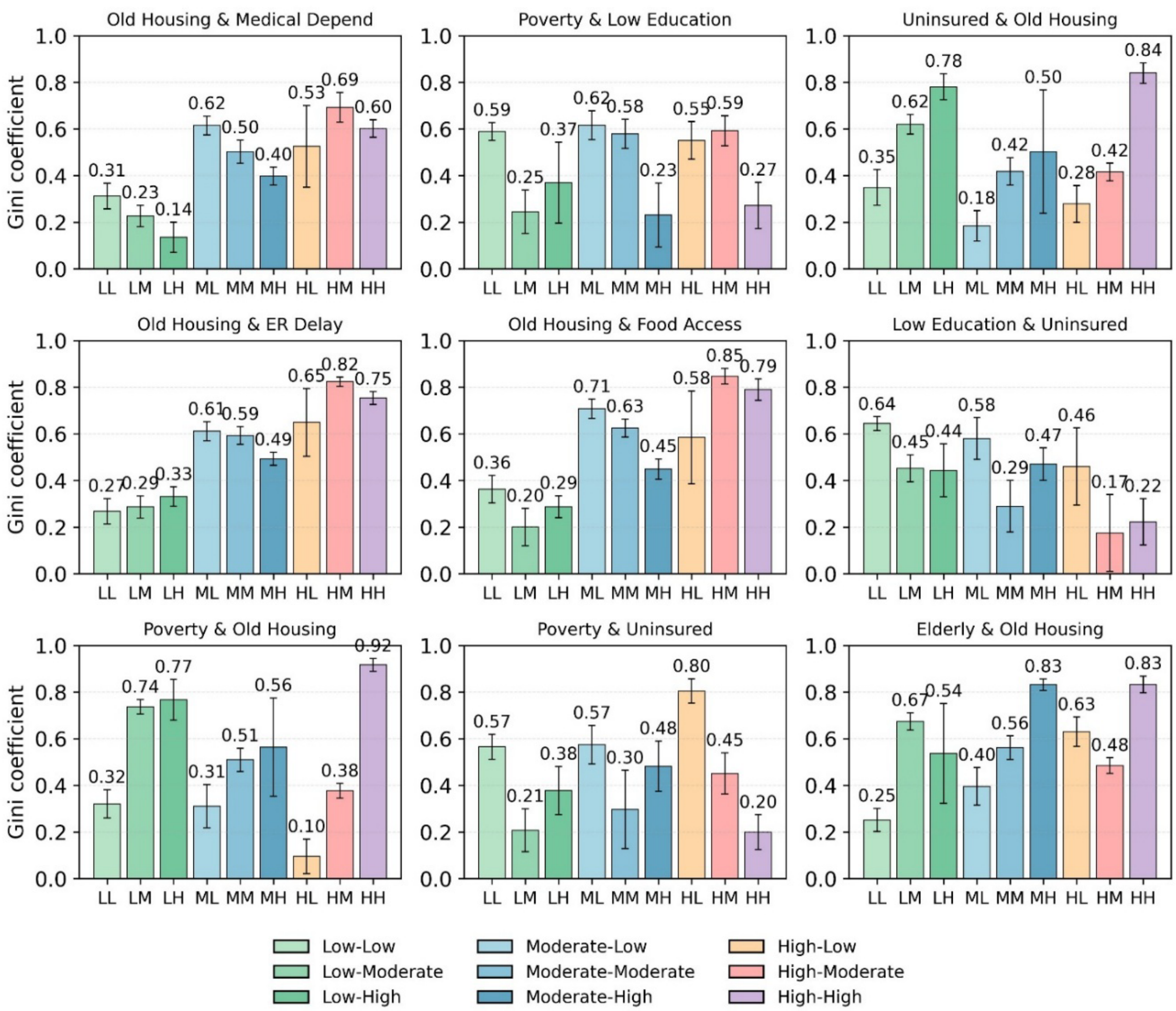

Figure S-13. Uncertainty in Gini-Based Spatial Heterogeneity Estimates. Gini coefficients for CDHW-day occurrence across selected paired socioeconomic vulnerability categories. Error bars show bootstrap standard errors estimated from 100 county-level resamples.

<u>Interpretation for Figure S-13.</u> This figure evaluates the robustness of Gini-based spatial heterogeneity estimates for CDHW-day occurrence across selected paired socioeconomic vulnerability categories. Higher Gini values indicate that CDHW-day occurrence is more concentrated among a smaller subset of counties within that vulnerability grouping, while lower values indicate a more even distribution. The results show that several paired categories involving old housing, uninsured populations, poverty, food access, medical dependency, and delayed emergency access exhibit high Gini coefficients, especially in Moderate–High, High–Moderate, and High–High groupings. The relatively small bootstrap error bars in most categories suggest that these spatial heterogeneity patterns are stable under county-level resampling. Wider error bars in some groups indicate greater within-group variability or smaller sample sizes, but the broader pattern remains consistent: CDHW-day occurrence is more spatially uneven where multiple socioeconomic vulnerabilities overlap.

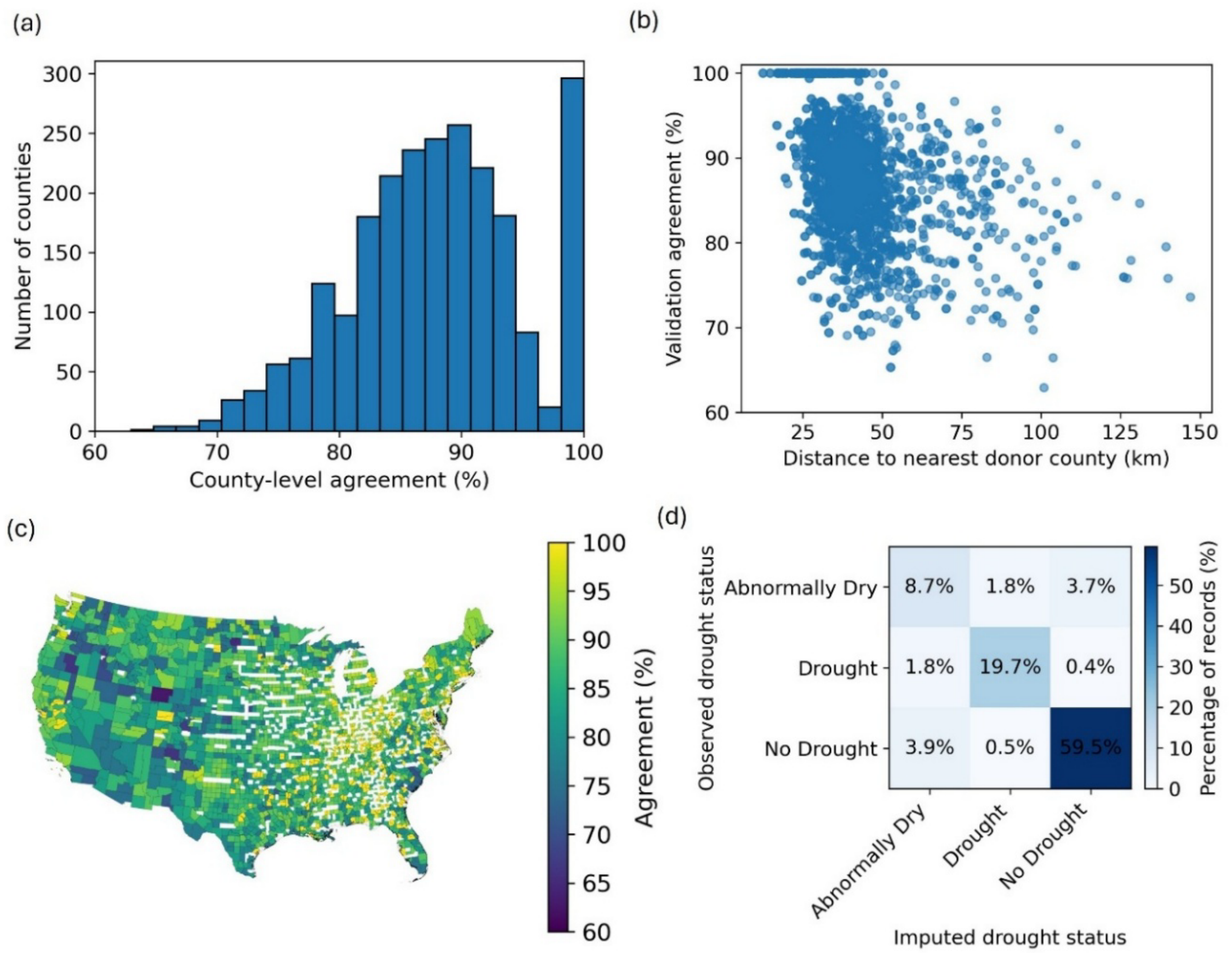

Figure S-14. Validation of the Voronoi-based nearest-neighbor imputation. (a) Distribution of county-level agreement from the leave-one-county-out validation. (b) Relationship between validation agreement and distance to the nearest donor county, defined as the nearest county with observed drought information used to reconstruct the withheld county. (c) Spatial distribution of county-level validation agreement. (d) Confusion matrix comparing observed and imputed drought categories.

Interpretation for Figure S-14. This figure evaluates the reliability of the Voronoi-based nearest-neighbor imputation method used to fill missing county-level drought information. The leave-one-county-out validation shows generally high agreement between observed and imputed drought categories, with most counties achieving agreement above 80%. The distance-based comparison indicates that validation agreement remains relatively high for most donor distances, although some decline and greater variability occur where the nearest donor county is farther away. The spatial map further shows that high agreement is widespread across the CONUS, while lower agreement appears in more localized areas, suggesting that imputation performance varies geographically. The confusion matrix indicates that the method performs best for the broader Drought and No Drought categories, while Abnormally Dry conditions show comparatively lower agreement, likely because this transitional category is more difficult to distinguish. Overall, the validation supports the use of Voronoi-based nearest-neighbor imputation for reconstructing missing drought status, while also showing that uncertainty is greater in areas with larger donor distances or transitional drought conditions.

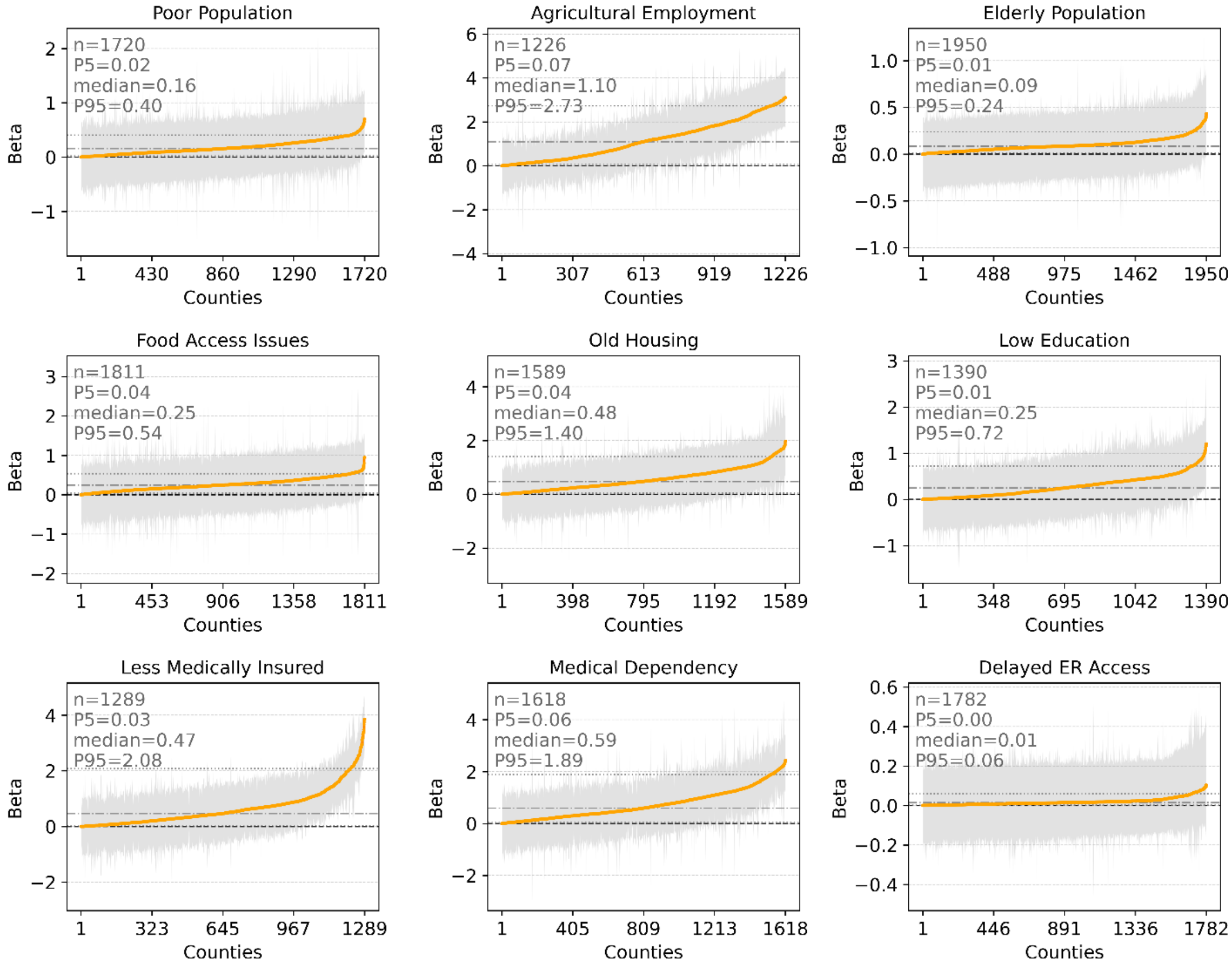

Figure S-15. County-level uncertainty in positive Bayesian coefficient estimates. Orange lines show county-level posterior mean beta estimates for counties with positive coefficients, sorted by beta magnitude. Light-gray bands show the corresponding lower–upper Bayesian credible interval bounds.

Interpretation for Figure S-15. This figure shows how uncertain the positive county-level Bayesian coefficient estimates are. The orange line gives the posterior mean coefficient for each county, sorted from smaller to larger values. The gray band gives the credible interval around each estimate. The results show that positive associations are not the same everywhere. Some variables, such as Agricultural Employment, Old Housing, Less Medically Insured, and Medical Dependency, have larger positive beta values in the upper range, suggesting stronger positive associations in selected counties. Other variables, such as Delayed ER Access and Elderly Population, show much smaller beta values, indicating weaker positive associations. Wider gray bands indicate higher uncertainty, especially for counties with larger beta values.

Supplementary Table

Table 1. Socioeconomic vulnerability variables and descriptions.

Vulnerability	Variables	Descriptions
Economic Vulnerability	Poor Population	% Below Poverty Line
	Agricultural Employment	% Employed in Agriculture
Demographic Sensitivity	Elderly Population	% Elderly Population (Aged 65+)
	Low Education	% Without High School Diploma
Infrastructure and Built Environment Vulnerability	Old Housing	% of Houses built before 1950
	Food Access Issue	% Population in Food Desert
Health & Medical Vulnerability	Uninsured Population	% Without Health Insurance
	Medical Dependency	% With Medical Equipment Dependency
	Delayed ER Access	% Population Without Timely Emergency Room (ER) Access

Table 2. Regional associations between changes in CDHW occurrences and shifts in population

Region	n	R^2	Adj R^2	CDHW β (sig)	Poor β (sig)	AgriEmp β (sig)
Central	405	0.507	0.502	−1.6322***	−0.8927***	−0.8580***
East North Central	437	0.426	0.422	−1.1374***	−0.9254***	−0.8150***
Northeast	217	0.248	0.238	+0.7303*	−0.4977**	−1.2697***
Northwest	198	0.220	0.208	−0.9422 (NS)	−0.5333**	−0.2984**
South	379	0.422	0.419	+0.3266 (NS)	−0.9592***	−0.8552***
Southeast	589	0.456	0.453	+2.1368***	−1.1685***	−0.6248***
Southwest	213	0.549	0.518	−1.0051 (NS)	−0.7417***	−0.8579***
West	168	0.271	0.257	−1.6679*	−0.4884*	−0.4747***
West North Central	503	0.155	0.151	−0.6148**	−0.5522***	−0.3189***

Table 3. | Variance inflation factor diagnostics for regional regression predictors.

**Region ID**	**n**	**VIF: CDHW**	**VIF: Poor**	**VIF: AgriEmp**
1	217	1.00	2.18	2.18
2	437	1.03	2.26	2.28
3	503	1.08	2.48	2.59
4	589	1.03	2.04	2.08
5	405	1.16	2.34	2.47
6	213	1.00	2.86	2.87
7	379	1.03	1.68	1.72
8	168	1.02	2.39	2.36
9	198	1.04	2.27	2.32

Data, Materials, and Software Availability statement

The datasets used in this study are publicly available from multiple sources. Weekly drought conditions were obtained from the U.S. Drought Monitor (USDM), accessible at https://datadryad.org/dataset/doi:10.5061/dryad.g1jwstqw7. Daily maximum and minimum 2-meter air temperature datasets were obtained from the Climate Prediction Center (CPC), accessible at https://psl.noaa.gov/data/gridded/data.cpc.globaltemp.html. Socioeconomic variables were sourced from the USDA Atlas of Rural and Small-Town America (https://www.ers.usda.gov/data-products/atlas-of-rural-and-small-town-america), the U.S. Department of Health and Human Services emPOWER Program (https://empowermap.hhs.gov), and the U.S. Census Bureau's American Community Survey (ACS), accessible via https://data.census.gov. Hospital facility data were obtained from the CMS Hospital General Information dataset, accessible at https://data.cms.gov/provider-data/search?pagesize=50&theme=Hospitals. All analyses were performed using Python and R, and scripts for modeling are available from the corresponding author upon reasonable request.

SI References

1. J. M. Eberth, *et al.*, The Problem Of The Color Line: Spatial Access To Hospital Services For Minoritized Racial And Ethnic Groups: Study examines spatial access to hospital services for minoritized racial and ethnic groups. *Health Affairs* **41**, 237–246 (2022).

2. J. Besag, Spatial interaction and the statistical analysis of lattice systems. *Journal of the Royal Statistical Society: Series B (Methodological)* **36**, 192–225 (1974).

3. W. R. Tobler, A computer movie simulating urban growth in the Detroit region. *Economic geography* **46**, 234–240 (1970).

4. D. Simpson, H. Rue, A. Riebler, T. G. Martins, S. H. Sørbye, Penalising model component complexity: A principled, practical approach to constructing priors. (2017).

5. S. H. Sørbye, H. Rue, Scaling intrinsic Gaussian Markov random field priors in spatial modelling. *Spatial Statistics* **8**, 39–51 (2014).

6. G.-A. Fuglstad, D. Simpson, F. Lindgren, H. Rue, Constructing priors that penalize the complexity of Gaussian random fields. *Journal of the American Statistical Association* **114**, 445–452 (2019).

7. H. Rue, S. Martino, N. Chopin, Approximate Bayesian inference for latent Gaussian models by using integrated nested Laplace approximations. *Journal of the Royal Statistical Society Series B: Statistical Methodology* **71**, 319–392 (2009).

8. V. Gómez-Rubio, *Bayesian inference with INLA* (Chapman and Hall/CRC, 2020).

9. B. Zhang, S. Wang, J. Zscheischler, H. Moradkhani, Higher Exposure of Poorer People to Emerging Weather Whiplash in a Warmer World. *Geophysical Research Letters* **50**, e2023GL105640 (2023).
